# Supplementary material for: Prevalence and correlates of disability in Latin America and the Caribbean: Evidence from 8 national censuses
Source: PLoS One. 2021 Oct 27;16(10):e0258825. doi: 10.1371/journal.pone.0258825 (PMC8550602; doi:10.1371/journal.pone.0258825)
Supplement: S1 Table — (PDF) [file pone.0258825.s001.pdf]

Table S1: **GDP per Capita and Population Structure**

Country	GDP per capita (constant 2010 US$) 2010	% population		
		0-19 years	20-24 years	55+ years
Brazil	11286	33.6	51.9	14.5
Costa Rica	8227.1	33.5	51.4	15.1
Dominican Republic	5555.4	40.8	47.1	12
Ecuador	4633.6	40.8	47.1	12.2
Mexico	9271.4	39.2	48.3	12.5
Panama	8082	38	48.6	13.4
Trinidad and Tobago	16683.4	28.2	54	17.7
Uruguay	11992	30.1	46.3	23.6

Source: authors' estimations based on data by United Nations Economic Commission for Latin America and the Caribbean (CEPAL).
