# Supplementary material for: Prevalence and correlates of disability in Latin America and the Caribbean: Evidence from 8 national censuses
Source: PLoS One. 2021 Oct 27;16(10):e0258825. doi: 10.1371/journal.pone.0258825 (PMC8550602; doi:10.1371/journal.pone.0258825)
Supplement: S2 Table — (PDF) [file pone.0258825.s002.pdf]

Table S2: Sampling Method by Sample

**Sample**	Pop . Fraction	Systematic sampling	Explicit stratification	Geographic clustering	Household clustering	Differential weighting
Brazil 2010	5%	.	X	.	X	X
Costa Rica 2011	10%	X	.	.	X	.
Dominican Rep. 2010	10%	X	.	.	X	.
Ecuador 2010	10%	X	.	.	X	.
Mexico 2010	10%	.	.	X	X	X
Panama 2010	10%	X	.	.	X	.
Trinidad & Tob . 2011	8.8%	X	.	.	X	.
Uruguay 2011	10%	X	.	.	X	.

Samples are provided by Minnesota Population Center (IPUMS International, 2018) from censuses and surveys collected by National Statistics Offices in each country. Brazil: Institute of Geography and Statistics; Costa Rica: National Institute of Statistics and Censuses; Dominican Republic: National Statistics Office; Ecuador: National Institute of Statistics and Censuses; Mexico: National Institute of Statistics, Geography, and Informatics; Panama: Census and Statistics Directorate; Trinidad and Tobago: Central Statistical Office; Uruguay: National Institute of Statistics.
