# Supplementary material for: Prevalence and correlates of disability in Latin America and the Caribbean: Evidence from 8 national censuses
Source: PLoS One. 2021 Oct 27;16(10):e0258825. doi: 10.1371/journal.pone.0258825 (PMC8550602; doi:10.1371/journal.pone.0258825)
Supplement: S3 Table — (PDF) [file pone.0258825.s003.pdf]

Table S3.1: Missing vs. Non-Missing: Descriptive Stats - Dominican Republic 2010

	Non-Missing			Missing			
	N (sample)	Mean	SD	N (sample)	Mean	SD	Diff.
Age	876897	30.29	19.66	11653	29.82	19.99	-0.470**
Female	876897	0.50	0.50	11653	0.49	0.50	-0.009**
Emp. Status: NIU	876897	0.14	0.35	11653	0.16	0.37	0.017***
Emp. Status: Employed	876897	0.34	0.47	11653	0.28	0.45	-0.060***
Emp. Status: Unemployed	876897	0.06	0.24	11653	0.04	0.20	-0.020***
Emp. Status: Inactive	876897	0.44	0.50	11653	0.35	0.48	-0.084***
Emp. Status: Missing	876897	0.01	0.12	11653	0.16	0.37	0.146***
Education: NIU	876897	0.00	0.00	11653	0.00	0.00	0.000
Education: < Primary	876897	0.44	0.50	11653	0.48	0.50	0.036***
Education: Primary	876897	0.32	0.47	11653	0.29	0.45	-0.039***
Education: Secondary	876897	0.17	0.38	11653	0.18	0.38	0.007*
Education: University	876897	0.06	0.24	11653	0.06	0.23	-0.004*

Table S3.1 shows descriptive statistics for respondent who were assigned a disability status and those who were considered as missing, separately. The "Diff" column is the coefficient of a regression of a "missing" indicator variable (taking value one for those with missing data on disability) on the variable. Stars indicate whether the coefficient is statistically significant. * $p < 0.10$, ** $p < 0.05$, *** $p < 0.01$.

Table S3.2: Missing vs. Non-Missing: Descriptive Stats - Trinidad and Tobago 2011

	Non-Missing			Missing			
	N (sample)	Mean	SD	N (sample)	Mean	SD	Diff.
Age	102979	35.69	20.16	8895	36.24	20.65	0.557
Female	102979	0.50	0.50	9083	0.48	0.50	-0.024***
Emp. Status: NIU	102979	0.17	0.37	9083	0.27	0.45	0.106***
Emp. Status: Employed	102979	0.47	0.50	9083	0.10	0.30	-0.370***
Emp. Status: Unemployed	102979	0.03	0.18	9083	0.01	0.08	-0.029***
Emp. Status: Inactive	102979	0.31	0.46	9083	0.07	0.25	-0.243***
Emp. Status: Missing	102979	0.02	0.14	9083	0.56	0.50	0.535***
Education: NIU	102979	0.00	0.00	9083	0.11	0.31	0.111***
Education: < Primary	102979	0.22	0.41	9083	0.21	0.41	-0.007
Education: Primary	102979	0.26	0.44	9083	0.22	0.42	-0.040***
Education: Secondary	102979	0.47	0.50	9083	0.41	0.49	-0.060***
Education: University	102979	0.04	0.19	9083	0.04	0.19	0.001
Education: University	102979	0.02	0.13	9083	0.01	0.11	-0.004**

Table S3.2 shows descriptive statistics for respondent who were assigned a disability status and those who were considered as missing, separately. The "Diff" column is the coefficient of a regression of a "missing" indicator variable (taking value one for those with missing data on disability) on the variable. Stars indicate whether the coefficient is statistically significant. * $p < 0.10$, ** $p < 0.05$, *** $p < 0.01$.

Table S3.3: Missing vs. Non-Missing: Descriptive Stats - Uruguay 2011

	Non-Missing			Missing			
	N (sample)	Mean	SD	N (sample)	Mean	SD	Diff.
Age	304188	37.52	22.38	11194	40.01	24.57	2.496***
Female	304188	0.52	0.50	11194	0.49	0.50	-0.030***
Emp. Status: NIU	304188	0.14	0.34	11194	0.09	0.29	-0.044***
Emp. Status: Employed	304188	0.49	0.50	11194	0.00	0.04	-0.484***
Emp. Status: Unemployed	304188	0.03	0.18	11194	0.00	0.00	-0.033***
Emp. Status: Inactive	304188	0.35	0.48	11194	0.00	0.03	-0.344***
Emp. Status: Missing	304188	0.00	0.00	11194	0.91	0.29	0.905***
Education: NIU	304188	0.01	0.12	11194	0.01	0.10	-0.003***
Education: < Primary	304188	0.23	0.42	11194	0.29	0.45	0.061***
Education: Primary	304188	0.52	0.50	11194	0.22	0.41	-0.300***
Education: Secondary	304188	0.20	0.40	11194	0.03	0.17	-0.164***
Education: University	304188	0.05	0.21	11194	0.00	0.00	-0.046***
Education: University	304188	0.00	0.00	11194	0.45	0.50	0.453

Table S3.3 shows descriptive statistics for respondent who were assigned a disability status and those who were considered as missing, separately. The "Diff" column is the coefficient of a regression of a "missing" indicator variable (taking value one for those with missing data on disability) on the variable. Stars indicate whether the coefficient is statistically significant. * $p < 0.10$, ** $p < 0.05$, *** $p < 0.01$.
