# Supplementary material for: Prevalence and correlates of disability in Latin America and the Caribbean: Evidence from 8 national censuses
Source: PLoS One. 2021 Oct 27;16(10):e0258825. doi: 10.1371/journal.pone.0258825 (PMC8550602; doi:10.1371/journal.pone.0258825)
Supplement: S4 Table — (PDF) [file pone.0258825.s004.pdf]

Table S4: Wording of the Surveys' Disability-related Questions and Response Categories.

Sample	Question and Response Categories
Brazil 2010	**Question:** Do you have any permanent difficulty in seeing? (If you wear glasses or contact lenses, make your evaluation while wearing them). **Responses:** [] 1 Yes, cannot do it at all; [] 2 Yes, major trouble; [] 3 Yes, some difficulty; [] 4 No
	**Q:** Do you have any permanent difficulty in hearing? (If you wear a rearing aid, make your evaluation while wearing it)<br>**R:** [] 1 Yes, cannot do it at all; [] 2 Yes, major trouble; [] 3 Yes, some difficulty; [] 4 No
	**Q:** Do you have any permanent difficulty in walking or climbing stairs? (If you use a prosthesis, cane or assistive device, make your evaluation while using it) **R:** [] 1 Yes, cannot do it at all; [] 2 Yes, major trouble; [] 3 Yes, some difficulty; [] 4 No
	**Q:** Do you have any permanent mental or intellectual disability that limits you in your daily activities such as working, going to school, playing, etc.? **R:** [] 1 Yes; [] 2 No
Costa Rica 2011	**Q:** Does [the respondent] have a permanent limitation? (You can mark multiple options)<br>**R:** [] 1 Seeing, even with glasses or contacts on; [] 2 Hearing; [] 3 Speaking; [] 4 Walking or going up steps; [] 5 Using arms or hands; [] 6 Intellectually (retarded, Down's syndrome, other); [] 7 Mentally (bipolar, schizophrenic, other); [] 8 None of the above
Dominican Republic 2010	**Q:** Does [the respondent] have permanent difficulty with any of the following? a. Seeing, even if he/she uses glasses?<br>**R:** [] 1 Yes; [] 2 No
	**Q:** Hearing, even if uses hearing aid? **R:** [] 1 Yes; [] 2 No
	**Q:** Walking or climbing stairs? **R:** [] 1 Yes; [] 2 No
	**Q:** Moving one or both arms? **R:** [] 1 Yes; [] 2 No
	**Q:** Moving or both legs? **R:** [] 1 Yes; [] 2 No
	**Q:** Remembering or concentrating? **R:** [] 1 Yes; [] 2 No
	**Q:** Grasping objects and/or opening containers with his/her hands? **R:** [] 1 Yes; [] 2 No
	**Q:** Speaking? **R:** [] 1 Yes; [] 2 No
	**Q:** Is he/she mute? **R:** [] 1 Yes; [] 2 No
	**Q:** Does he/she have mental problems? **R:** [] 1 Yes; [] 2 No
	**Q:** Is he/she or missing one or both legs? **R:** [] 1 Yes; [] 2 No
	**Q:** Is he/she or missing one or both arms? **R:** [] 1 Yes; [] 2 No

The wording of the questions (translated to English) by Minnesota Population Center (IPUMS International, 2018). The version in their original language is available in their website. Surveys were collected by National Statistics Offices in each country. Brazil: Institute of Geography and Statistics; Costa Rica: National Institute of Statistics and Censuses; Dominican Republic: National Statistics Office.

Table S4 (Continued): Wording of the Surveys' Disability-related Questions and Response Categories.

Sample	Question and Response Categories
Ecuador 2010	**Q:** Does [the respondent] have a permanent disability that has lasted for more than one year? **R:** [] 1 Yes; [] 2 No (skip [next question]); [] 9 No response (skip [next question]).
	**Q:** The disability of [the respondent] is: **R:** [] 1 Intellectual? (mental retardation); [] 2 Physical/motor? (paralysis and amputations); [] 3 Visual? (blindness); [] 4 Auditory? (deafness); [] 5 Mental? (psychiatric illnesses, craziness)
Mexico 2010	**Q:** Does [the respondent] have difficulty doing the following activities in his/her daily life: Read all the options and circle those with an affirmative answer. **R:** [] 1 Walking, moving, going up and down [the stairs]; [] 2 Seeing, even when using glasses; [] 3 Speaking, communicating or conversing; [] 4 Hearing, even when using a hearing aid; [] 5 Getting dressed, bathing or eating; [] 6 Paying attention or learning simple things; [] 7 Has any mental limitations; [] 8 This person does not have any physical or mental difficulties (skip [next question])
	**Q:** [The respondent] has difficulty in (answer to previous question): For each option circled in the [previous question] read the options and circle only one reason code. **R:** [] 1 Because s/he was born that way; [] 2 Due to an illness; [] 3 Due to an accident; [] 4 Due to advanced age; [] 5 For another reason
Panama 2010	**Q:** Even with hearing aids for loss of hearing, do you have trouble hearing? **R:** [] 1 Yes; [] 2 No
	**Q:** Even using eyeglasses, do you have difficulty seeing? **R:** [] 1 Yes; [] 2 No
	**Q:** Do you have any permanent difficulty for walking or getting around? **R:** [] 1 Yes; [] 2 No
	**Q:** Do you have any permanent difficulty for using your arms and/or hands? **R:** [] 1 Yes; [] 2 No
	**Q:** Do you have any permanent difficulty for speaking or communicating? **R:** [] 1 Yes; [] 2 No
	**Q:** Do you have any permanent learning difficulty? **R:** [] 1 Yes; [] 2 No
	**Q:** What type of physical or mental disability do you have? [] 1 Blindness; [] 2 Deafness; [] 3 Mental retardation; [] 4 Cerebral paralysis; [] 5 Physical deficiency; [] 6 Mental problems; [] 7 Other (Specify); [] 8 None.

The wording of the questions (translated to English) by Minnesota Population Center (IPUMS International, 2018). The version in their original language is available in their website. Surveys were collected by National Statistics Offices in each country. Ecuador: National Institute of Statistics and Censuses; Mexico: National Institute of Statistics, Geography, and Informatics; Panama: Census and Statistics Directorate.

Table S4 (Continued): Wording of the Surveys' Disability-related Questions and Response Categories.

Sample	Question and Response Categories
Trinidad and Tobago 2011	**Q:** Does (N) [the respondent] suffer from any long-standing disability?<br>**R:** [] 1 Yes; [] 2 No – Skip [next 3 questions]; [] 9 Not stated – Skip [next 3 questions]
	**Q:** Does the long-standing disability prevent (N) [the respondent] from doing any of the following? [Respondents indicate both the activity and its level of difficulty]<br>Activities:<br>**R:** [] 01 Seeing even if wearing glasses?; [] 02 Hearing even if using hearing aid?; [] 03 Walking, climbing steps? (Moving/being mobile); [] 04 Remembering or concentrating? (Learning); [] 05 Gripping?; [] 06 Speaking and understanding because of a physical, mental or emotional health condition?<br>Level:<br>[] a. Yes - some difficulty; [] b. Yes - a lot of difficulty; [] c. Cannot do at all
	**Q:** Does the long-standing disability prevent (N) [the respondent] from doing any of the following? [Respondents indicate both the activity and its level of difficulty]<br>**R:** [] a. Yes - some difficulty; [] b. Yes - a lot of difficulty; [] c. Cannot do at all
	**Q:** Does any of (N's) [the respondent's] disabilities or conditions affect (N) [the respondent] from performing any of the following?<br>**R:** [] 01 Taking care of himself/herself (TCH); [] 02 Getting around within the home (GAH); [] 03 Going outside the home (GOH); [] 04 Working at a job or business (WB); [] 05 Undertaking educational activities (UEA); [] 06 Participating in social activities (PSA); [] 07 No effect (NE)
Uruguay 2011	**Q:** Does [the respondent] have permanent difficulty with: Seeing, even if using glasses or lenses<br>**R:** [] 1 Does not have any difficulty; [] 2 Yes, some difficulty; [] 3 Yes, much difficulty; [] 4 Yes, [the respondent] cannot do it
	**Q:** Hearing, even if using hearing aids<br>**R:** [] 1 Does not have any difficulty; [] 2 Yes, some difficulty; [] 3 Yes, much difficulty; [] 4 Yes, [the respondent] cannot do it
	**Q:** Walking or going up stairs<br>**R:** [] 1 Does not have any difficulty; [] 2 Yes, some difficulty; [] 3 Yes, much difficulty; [] 4 Yes, [the respondent] cannot do it
	**Q:** Understanding and/or learning (For people 6 years or older)<br>**R:** [] 1 Does not have any difficulty; [] 2 Yes, some difficulty; [] 3 Yes, much difficulty; [] 4 Yes, [the respondent] cannot do it

The wording of the questions (translated to English) by Minnesota Population Center (IPUMS International, 2018). The version in their original language is available in their website. Surveys were collected by National Statistics Offices in each country. Trinidad and Tobago: Central Statistical Office; Uruguay: National Institute of Statistics.

Table S4 (Continued): Wording of the Surveys' Disability-related Questions and Response Categories.

Sample	Question and Response Categories
The Washington Group Short Set on Functioning (WG-SS)	The next questions ask about difficulties you may have doing certain activities because of a HEALTH PROBLEM. **Q:** [Do/Does] [you/he/she] have difficulty seeing, even if wearing glasses? Would you say... **R:** [] 1. No difficulty; [] 2. Some difficulty; [] 3. A lot of difficulty; [] 4. Cannot do at all; [] 7. Refused; [] 9. Don't know
	**Q:** [Do/Does] [you/he/she] have difficulty hearing, even if using a hearing aid(s)? Would you say... **R:** [] 1. No difficulty; [] 2. Some difficulty; [] 3. A lot of difficulty; [] 4. Cannot do at all; [] 7. Refused; [] 9. Don't know
	**Q:** [Do/Does] [you/he/she] have difficulty walking or climbing steps? Would you say... **R:** [] 1. No difficulty; [] 2. Some difficulty; [] 3. A lot of difficulty; [] 4. Cannot do at all; [] 7. Refused; [] 9. Don't know
	**Q:** [Do/does] [you/he/she] have difficulty remembering or concentrating? Would you say... **R:** [] 1. No difficulty; [] 2. Some difficulty; [] 3. A lot of difficulty; [] 4. Cannot do at all; [] 7. Refused; [] 9. Don't know
	**Q:** [Do/does] [you/he/she] have difficulty with self-care, such as washing all over or dressing? Would you say... **R:** [] 1. No difficulty; [] 2. Some difficulty; [] 3. A lot of difficulty; [] 4. Cannot do at all; [] 7. Refused; [] 9. Don't know
	**Q:** Using [your/his/her] usual language, [do/does] [you/he/she] have difficulty communicating, for example understanding or being understood? Would you say... **R:** [] 1. No difficulty; [] 2. Some difficulty; [] 3. A lot of difficulty; [] 4. Cannot do at all; [] 7. Refused; [] 9. Don't know

The Washington Group Short Set on Functioning (WG-SS) was downloaded from https://www.washingtongroup-disability.com/question-sets/wg-short-set-on-functioning-wg-ss/ (accessed on August 31st, 2021).
