# Supplementary material for: Prevalence and correlates of disability in Latin America and the Caribbean: Evidence from 8 national censuses
Source: PLoS One. 2021 Oct 27;16(10):e0258825. doi: 10.1371/journal.pone.0258825 (PMC8550602; doi:10.1371/journal.pone.0258825)
Supplement: S5 Table — (PDF) [file pone.0258825.s005.pdf]

Table S5: Prevalence of Disability by Country and Gender: Estimates (Ages 15 and older).

	Both Sexes			Men			Women		
	Est.	95% C.I.		Est.	95% C.I.		Est.	95% C.I.	
Brazil	29.1	[29.1	29.2]	25.9	[25.8	25.9]	32.1	[32.1	32.2]
Costa Rica	12.8	[12.6	12.9]	12.5	[12.3	12.7]	13.0	[12.8	13.2]
Dominican R.	16.3	[16.2	16.4]	13.9	[13.8	14.1]	18.6	[18.5	18.7]
Ecuador	6.32	[6.27	6.38]	6.87	[6.80	6.95]	5.79	[5.73	5.86]
Mexico	6.58	[6.52	6.64]	6.60	[6.49	6.70]	6.56	[6.50	6.62]
Panama	10.2	[10.1	10.3]	9.76	[9.59	9.94]	10.6	[10.5	10.8]
Trinidad & T.	5.12	[4.96	5.28]	4.96	[4.75	5.18]	5.27	[5.05	5.49]
Uruguay	19.3	[19.2	19.5]	16.2	[16.0	16.4]	22.1	[21.9	22.4]

Source: authors' estimations based on data provided by Minnesota Population Center (IPUMS International, 2018) from censuses collected by National Statistics Offices in each country. Estimates for Brazil, Dominican Republic, Ecuador, Mexico, and Panama refer to the year 2010. Estimates for Costa Rica, Trinidad and Tobago, and Uruguay refer to the year 2011. Estimates consider individuals aged 15 years and older.
