# Supplementary material for: Prevalence and correlates of disability in Latin America and the Caribbean: Evidence from 8 national censuses
Source: PLoS One. 2021 Oct 27;16(10):e0258825. doi: 10.1371/journal.pone.0258825 (PMC8550602; doi:10.1371/journal.pone.0258825)
Supplement: S6 Table — (PDF) [file pone.0258825.s006.pdf]

Table S6: Prevalence of 'Severe' Disability by Country and Gender: Estimates (Ages 3 and older).

	Both Sexes			Men			Women		
	Est.	95% C.I.		Est.	95% C.I.		Est.	95% C.I.	
Brazil	6.92	[6.90	6.94]	6.28	[6.25	6.30]	7.53	[7.50	7.56]
Trinidad & T.	2.38	[2.28	2.48]	2.36	[2.23	2.50]	2.40	[2.26	2.54]
Uruguay	5.00	[4.92	5.09]	4.01	[3.90	4.11]	5.91	[5.79	6.03]

Source: authors' estimations based on data provided by Minnesota Population Center (IPUMS International, 2018) from censuses collected by National Statistics Offices in each country. Estimates for Brazil refer to the year 2010. Estimates for Trinidad and Tobago and Uruguay refer to the year 2011. The survey questions in Brazil, Trinidad and Tobago, and Uruguay inquire about the degree of the difficulty or limitation. The prevalence rates reported in this table consider only individuals who responded having at least a "a lot of difficulty". It excludes individuals who responded having a "some difficulty". Estimates consider individuals aged 3 years and older.
