# Supplementary material for: Prevalence and correlates of disability in Latin America and the Caribbean: Evidence from 8 national censuses
Source: PLoS One. 2021 Oct 27;16(10):e0258825. doi: 10.1371/journal.pone.0258825 (PMC8550602; doi:10.1371/journal.pone.0258825)
Supplement: S7 Table — (PDF) [file pone.0258825.s007.pdf]

Table S7: Prevalence of Disability by Country and Sex: Estimates by Age Group

	Both Sexes			Men			Women		
Ages 3-5	Est.	95% C.I.		Est.	95% C.I.		Est.	95% C.I.	
Brazil	3.83	[3.76	3.89]	4.06	[3.96	4.16]	3.59	[3.49	3.68]
Costa Rica	2.21	[2.01	2.42]	2.46	[2.18	2.78]	1.94	[1.67	2.23]
Dominican R.	1.90	[1.78	2.02]	2.20	[2.02	2.38]	1.59	[1.44	1.75]
Ecuador	1.73	[1.64	1.82]	1.90	[1.78	2.04]	1.55	[1.43	1.67]
Mexico	1.28	[1.23	1.33]	1.48	[1.40	1.57]	1.07	[1.01	1.14]
Panama	1.10	[0.95	1.26]	1.14	[0.93	1.37]	1.06	[0.86	1.29]
Trinidad & T.	0.79	[0.55	1.09]	1.17	[0.77	1.69]	0.40	[0.18	0.76]
Uruguay	1.99	[1.75	2.26]	2.12	[1.78	2.51]	1.86	[1.53	2.22]
Ages 6-17	Est.	95% C.I.		Est.	95% C.I.		Est.	95% C.I.	
Brazil	10.5	[10.5	10.6]	9.75	[9.68	9.82]	11.3	[11.2	11.4]
Costa Rica	4.52	[4.38	4.67]	4.85	[4.65	5.07]	4.19	[4.00	4.39]
Dominican R.	4.22	[4.13	4.30]	4.03	[3.91	4.15]	4.40	[4.28	4.53]
Ecuador	2.64	[2.58	2.69]	2.94	[2.86	3.02]	2.33	[2.25	2.40]
Mexico	1.97	[1.94	2.01]	2.25	[2.20	2.31]	1.69	[1.64	1.73]
Panama	2.64	[2.52	2.77]	2.95	[2.77	3.13]	2.32	[2.17	2.49]
Trinidad & T.	1.40	[1.23	1.60]	1.43	[1.20	1.71]	1.37	[1.12	1.66]
Uruguay	7.59	[7.35	7.83]	7.98	[7.65	8.31]	7.18	[6.87	7.51]
Ages 18-55	Est.	95% C.I.		Est.	95% C.I.		Est.	95% C.I.	
Brazil	22.9	[22.8	22.9]	20.2	[20.2	20.3]	25.5	[25.4	25.5]
Costa Rica	8.50	[8.37	8.62]	8.51	[8.35	8.68]	8.48	[8.32	8.64]
Dominican R.	11.4	[11.3	11.5]	9.68	[9.57	9.80]	13.2	[13.1	13.3]
Ecuador	4.15	[4.10	4.20]	4.78	[4.71	4.85]	3.54	[3.48	3.60]
Mexico	3.37	[3.34	3.41]	3.71	[3.66	3.76]	3.06	[3.02	3.11]
Panama	5.97	[5.84	6.09]	5.77	[5.61	5.93]	6.16	[6.00	6.33]
Trinidad & T.	3.15	[3.00	3.30]	3.36	[3.16	3.57]	2.94	[2.75	3.14]
Uruguay	10.8	[10.6	11.0]	9.57	[9.36	9.78]	12.0	[11.8	12.2]
Ages 56+	Est.	95% C.I.		Est.	95% C.I.		Est.	95% C.I.	
Brazil	60.4	[60.3	60.6]	56.9	[56.8	57.1]	63.3	[63.2	63.5]
Costa Rica	33.3	[32.9	33.7]	32.5	[32.0	33.1]	34.0	[33.4	34.5]
Dominican R.	44.0	[43.7	44.3]	38.9	[38.5	39.3]	48.9	[48.5	49.3]
Ecuador	17.4	[17.2	17.6]	17.9	[17.7	18.2]	16.9	[16.7	17.2]
Mexico	22.7	[22.5	23.0]	21.7	[21.2	22.2]	23.7	[23.4	23.9]
Panama	29.3	[28.8	29.7]	28.1	[27.5	28.7]	30.5	[29.9	31.1]
Trinidad & T.	12.4	[11.9	12.9]	11.4	[10.7	12.1]	13.4	[12.7	14.0]
Uruguay	41.0	[40.6	41.4]	35.3	[34.8	35.9]	45.2	[44.8	45.7]

Source: authors' estimations based on data provided by Minnesota Population Center (IPUMS International, 2018) from censuses and surveys collected by National Statistics Offices in each country.
