# Supplementary material for: Prevalence and correlates of disability in Latin America and the Caribbean: Evidence from 8 national censuses
Source: PLoS One. 2021 Oct 27;16(10):e0258825. doi: 10.1371/journal.pone.0258825 (PMC8550602; doi:10.1371/journal.pone.0258825)
Supplement: S8 Table — (PDF) [file pone.0258825.s008.pdf]

Table S8.1: Prevalence of Disability by Country and Sex (Ages 3 and older): Estimates by Type of Disability

	Both Sexes			Men			Women		
Motor Disab.	Est.	95% C.I.		Est.	95% C.I.		Est.	95% C.I.	
Brazil	7.21	[7.19	7.23]	5.53	[5.51	5.56]	8.82	[8.79	8.85]
Costa Rica	3.91	[3.85	3.98]	3.75	[3.66	3.83]	4.07	[3.98	4.16]
Dominican R.	4.85	[4.80	4.90]	4.22	[4.16	4.28]	5.49	[5.43	5.56]
Ecuador	2.48	[2.45	2.50]	2.71	[2.67	2.75]	2.25	[2.21	2.28]
Mexico	3.15	[3.11	3.19]	2.95	[2.88	3.02]	3.34	[3.30	3.37]
Panama	3.16	[3.09	3.22]	3.03	[2.94	3.11]	3.29	[3.20	3.38]
Trinidad & T.	2.11	[2.01	2.20]	2.03	[1.91	2.15]	2.18	[2.05	2.32]
Uruguay	6.73	[6.63	6.82]	4.63	[4.52	4.74]	8.65	[8.51	8.79]
Vision Disab.	Est.	95% C.I.		Est.	95% C.I.		Est.	95% C.I.	
Brazil	19.6	[19.5	19.6]	16.7	[16.7	16.7]	22.3	[22.3	22.4]
Costa Rica	6.05	[5.96	6.14]	5.49	[5.38	5.60]	6.57	[6.46	6.69]
Dominican R.	8.92	[8.85	8.99]	7.00	[6.93	7.08]	10.9	[10.8	10.9]
Ecuador	1.35	[1.33	1.37]	1.41	[1.39	1.44]	1.29	[1.26	1.32]
Mexico	1.47	[1.44	1.51]	1.46	[1.38	1.53]	1.49	[1.47	1.51]
Panama	4.94	[4.85	5.02]	4.40	[4.30	4.51]	5.47	[5.35	5.59]
Trinidad & T.	1.73	[1.64	1.82]	1.51	[1.41	1.62]	1.95	[1.83	2.08]
Uruguay	10.2	[10.1	10.3]	7.98	[7.84	8.13]	12.3	[12.1	12.4]
Hearing Disab.	Est.	95% C.I.		Est.	95% C.I.		Est.	95% C.I.	
Brazil	5.30	[5.28	5.32]	5.47	[5.44	5.49]	5.14	[5.12	5.17]
Costa Rica	1.69	[1.65	1.74]	1.81	[1.75	1.88]	1.58	[1.53	1.64]
Dominican R.	1.77	[1.74	1.80]	1.72	[1.68	1.76]	1.82	[1.78	1.86]
Ecuador	0.87	[0.85	0.88]	0.94	[0.91	0.96]	0.80	[0.78	0.82]
Mexico	0.66	[0.64	0.67]	0.72	[0.71	0.74]	0.59	[0.57	0.61]
Panama	1.30	[1.26	1.34]	1.33	[1.28	1.39]	1.27	[1.21	1.32]
Trinidad & T.	0.52	[0.48	0.57]	0.50	[0.44	0.56]	0.55	[0.49	0.62]
Uruguay	3.89	[3.82	3.97]	3.64	[3.55	3.74]	4.13	[4.03	4.23]
Cognitive Disab.	Est.	95% C.I.		Est.	95% C.I.		Est.	95% C.I.	
Brazil	1.41	[1.40	1.42]	1.56	[1.54	1.57]	1.27	[1.26	1.28]
Costa Rica	0.85	[0.82	0.88]	0.98	[0.93	1.02]	0.74	[0.70	0.78]
Dominican R.	2.97	[2.93	3.01]	2.52	[2.47	2.57]	3.42	[3.37	3.48]
Ecuador	0.75	[0.73	0.76]	0.82	[0.80	0.84]	0.68	[0.66	0.70]
Mexico	0.24	[0.23	0.24]	0.26	[0.25	0.27]	0.22	[0.21	0.22]
Panama	1.52	[1.47	1.57]	1.60	[1.54	1.67]	1.44	[1.38	1.50]
Trinidad & T.	0.66	[0.61	0.71]	0.63	[0.56	0.70]	0.69	[0.62	0.77]
Uruguay	2.86	[2.79	2.93]	2.89	[2.80	2.99]	2.83	[2.74	2.92]

Source: authors' estimations based on data provided by Minnesota Population Center (IPUMS International, 2018) from censuses and surveys collected by National Statistics Offices in each country. Estimates for Brazil, Dominican Republic, Ecuador, Mexico, and Panama refer to the year 2010. Estimates for Costa Rica, Trinidad and Tobago, and Uruguay refer to the year 2011.

Table S8.2: Prevalence of Disability by Country and Sex (Ages 3-5): Estimates by Type of Disability

	Both Sexes			Men			Women		
Motor Disab.	Est.	95% C.I.		Est.	95% C.I.		Est.	95% C.I.	
Brazil	0.82	[0.79	0.86]	0.90	[0.85	0.94]	0.75	[0.71	0.80]
Costa Rica	0.55	[0.45	0.66]	0.55	[0.42	0.71]	0.55	[0.42	0.72]
Dominican R.	0.68	[0.61	0.75]	0.76	[0.66	0.87]	0.60	[0.51	0.70]
Ecuador	0.81	[0.75	0.87]	0.90	[0.81	0.99]	0.72	[0.65	0.81]
Mexico	0.48	[0.44	0.51]	0.50	[0.45	0.56]	0.45	[0.41	0.49]
Panama	0.41	[0.32	0.52]	0.37	[0.25	0.53]	0.45	[0.33	0.61]
Trinidad & T.	0.37	[0.22	0.59]	0.47	[0.24	0.85]	0.27	[0.098	0.58]
Uruguay	0.74	[0.60	0.91]	0.83	[0.62	1.08]	0.65	[0.46	0.88]

Vision Disab.	Est.	95% C.I.		Est.	95% C.I.		Est.	95% C.I.	
Brazil	2.06	[2.01	2.11]	2.09	[2.02	2.16]	2.02	[1.95	2.10]
Costa Rica	0.44	[0.36	0.55]	0.45	[0.33	0.60]	0.44	[0.32	0.58]
Dominican R.	0.52	[0.46	0.59]	0.61	[0.52	0.71]	0.44	[0.36	0.52]
Ecuador	0.28	[0.25	0.32]	0.29	[0.24	0.35]	0.27	[0.22	0.32]
Mexico	0.14	[0.12	0.15]	0.14	[0.11	0.16]	0.13	[0.12	0.16]
Panama	0.19	[0.13	0.27]	0.19	[0.10	0.32]	0.18	[0.10	0.29]
Trinidad & T.	0.13	[0.048	0.29]	0.086	[0.010	0.31]	0.18	[0.048	0.45]
Uruguay	0.95	[0.79	1.14]	0.98	[0.75	1.24]	0.92	[0.70	1.19]

Hearing Disab.	Est.	95% C.I.		Est.	95% C.I.		Est.	95% C.I.	
Brazil	0.95	[0.91	0.98]	1.03	[0.98	1.08]	0.86	[0.81	0.91]
Costa Rica	0.23	[0.17	0.30]	0.25	[0.16	0.36]	0.21	[0.12	0.32]
Dominican R.	0.28	[0.24	0.33]	0.29	[0.23	0.36]	0.27	[0.21	0.35]
Ecuador	0.21	[0.18	0.24]	0.25	[0.20	0.30]	0.17	[0.13	0.21]
Mexico	0.082	[0.069	0.098]	0.095	[0.073	0.12]	0.070	[0.055	0.088]
Panama	0.23	[0.16	0.31]	0.27	[0.16	0.40]	0.19	[0.11	0.30]
Trinidad & T.	0.044	[0.0053	0.16]	0.043	[0.0011	0.24]	0.044	[0.0011	0.25]
Uruguay	0.60	[0.47	0.75]	0.69	[0.50	0.92]	0.51	[0.35	0.72]

Cognitive Disab.	Est.	95% C.I.		Est.	95% C.I.		Est.	95% C.I.	
Brazil	0.64	[0.61	0.67]	0.75	[0.71	0.79]	0.52	[0.49	0.56]
Costa Rica	0.55	[0.45	0.66]	0.66	[0.51	0.83]	0.43	[0.31	0.58]
Dominican R.	0.57	[0.50	0.64]	0.68	[0.59	0.79]	0.44	[0.37	0.54]
Ecuador	0.51	[0.46	0.56]	0.54	[0.47	0.61]	0.48	[0.42	0.55]
Mexico	0.11	[0.096	0.12]	0.14	[0.12	0.16]	0.082	[0.066	0.100]
Panama	0.41	[0.32	0.52]	0.42	[0.30	0.59]	0.40	[0.28	0.55]
Trinidad & T.	0.13	[0.048	0.29]	0.22	[0.070	0.50]	0.044	[0.0011	0.25]

Source: authors' estimations based on data provided by Minnesota Population Center (IPUMS International, 2018) from censuses and surveys collected by National Statistics Offices in each country. Estimates for Brazil, Dominican Republic, Ecuador, Mexico, and Panama refer to the year 2010. Estimates for Costa Rica, Trinidad and Tobago, and Uruguay refer to the year 2011.

Table S8.3: Prevalence of Disability by Country and Sex (Ages 6-17): Estimates by Type of Disability

	Both Sexes			Men			Women		
Motor Disab.	Est.	95% C.I.		Est.	95% C.I.		Est.	95% C.I.	
Brazil	1.02	[1.00	1.03]	1.02	[1.00	1.04]	1.01	[0.99	1.03]
Costa Rica	0.61	[0.55	0.66]	0.67	[0.60	0.75]	0.54	[0.48	0.62]
Dominican R.	0.86	[0.82	0.90]	0.91	[0.85	0.97]	0.82	[0.76	0.87]
Ecuador	0.88	[0.85	0.91]	0.99	[0.95	1.04]	0.76	[0.72	0.80]
Mexico	0.52	[0.51	0.55]	0.58	[0.55	0.61]	0.47	[0.45	0.49]
Panama	0.52	[0.46	0.57]	0.58	[0.50	0.66]	0.45	[0.39	0.52]
Trinidad & T.	0.42	[0.33	0.53]	0.44	[0.31	0.60]	0.40	[0.27	0.56]
Uruguay	0.86	[0.79	0.94]	0.95	[0.84	1.06]	0.77	[0.67	0.88]

Vision Disab.	Est.	95% C.I.		Est.	95% C.I.		Est.	95% C.I.	
Brazil	7.99	[7.94	8.04]	6.93	[6.86	6.99]	9.08	[9.01	9.15]
Costa Rica	2.06	[1.96	2.16]	1.95	[1.82	2.09]	2.17	[2.03	2.32]
Dominican R.	2.12	[2.06	2.19]	1.63	[1.56	1.71]	2.63	[2.53	2.72]
Ecuador	0.59	[0.57	0.62]	0.63	[0.59	0.66]	0.56	[0.53	0.60]
Mexico	0.41	[0.40	0.43]	0.41	[0.38	0.43]	0.42	[0.39	0.44]
Panama	0.73	[0.67	0.80]	0.66	[0.59	0.75]	0.81	[0.72	0.91]
Trinidad & T.	0.40	[0.32	0.52]	0.39	[0.27	0.55]	0.41	[0.28	0.58]
Uruguay	3.12	[2.98	3.27]	2.56	[2.38	2.75]	3.71	[3.49	3.95]

Hearing Disab.	Est.	95% C.I.		Est.	95% C.I.		Est.	95% C.I.	
Brazil	1.69	[1.67	1.72]	1.78	[1.75	1.81]	1.61	[1.58	1.64]
Costa Rica	0.43	[0.39	0.48]	0.51	[0.45	0.58]	0.35	[0.30	0.41]
Dominican R.	0.49	[0.46	0.52]	0.52	[0.48	0.57]	0.46	[0.42	0.50]
Ecuador	0.37	[0.35	0.39]	0.41	[0.38	0.44]	0.32	[0.29	0.35]
Mexico	0.16	[0.15	0.17]	0.17	[0.16	0.19]	0.15	[0.14	0.16]
Panama	0.35	[0.30	0.39]	0.40	[0.34	0.47]	0.29	[0.24	0.35]
Trinidad & T.	0.18	[0.12	0.26]	0.15	[0.071	0.26]	0.21	[0.13	0.34]
Uruguay	0.81	[0.74	0.89]	0.94	[0.84	1.06]	0.68	[0.59	0.78]

Cognitive Disab.	Est.	95% C.I.		Est.	95% C.I.		Est.	95% C.I.	
Brazil	1.07	[1.05	1.09]	1.30	[1.28	1.33]	0.83	[0.81	0.85]
Costa Rica	1.22	[1.14	1.30]	1.40	[1.29	1.52]	1.04	[0.94	1.14]
Dominican R.	1.28	[1.24	1.33]	1.51	[1.44	1.58]	1.06	[1.00	1.12]
Ecuador	0.82	[0.79	0.85]	0.92	[0.88	0.97]	0.72	[0.68	0.76]
Mexico	0.33	[0.32	0.35]	0.42	[0.40	0.44]	0.25	[0.23	0.26]
Panama	1.49	[1.40	1.59]	1.82	[1.68	1.96]	1.16	[1.05	1.27]
Trinidad & T.	0.31	[0.22	0.41]	0.38	[0.26	0.54]	0.22	[0.13	0.38]
Uruguay	4.02	[3.84	4.21]	4.90	[4.64	5.17]	3.10	[2.89	3.32]

Source: authors' estimations based on data provided by Minnesota Population Center (IPUMS International, 2018) from censuses and surveys collected by National Statistics Offices in each country. Estimates for Brazil, Dominican Republic, Ecuador, Mexico, and Panama refer to the year 2010. Estimates for Costa Rica, Trinidad and Tobago, and Uruguay refer to the year 2011.

Table S8.4: Prevalence of Disability by Country and Sex (Ages 18-55): Estimates by Type of Disability

	Both Sexes			Men			Women		
Motor Disab.	Est.	95% C.I.		Est.	95% C.I.		Est.	95% C.I.	
Brazil	4.46	[4.44	4.48]	3.60	[3.58	3.63]	5.28	[5.25	5.31]
Costa Rica	2.32	[2.26	2.38]	2.53	[2.44	2.62]	2.12	[2.04	2.21]
Dominican R.	3.26	[3.21	3.32]	3.14	[3.07	3.21]	3.39	[3.32	3.47]
Ecuador	1.97	[1.94	2.00]	2.37	[2.32	2.42]	1.58	[1.54	1.62]
Mexico	1.64	[1.62	1.67]	1.75	[1.71	1.78]	1.55	[1.52	1.58]
Panama	1.64	[1.58	1.70]	1.75	[1.67	1.84]	1.53	[1.45	1.61]
Trinidad & T.	1.25	[1.17	1.35]	1.45	[1.32	1.59]	1.06	[0.94	1.18]
Uruguay	2.56	[2.48	2.64]	2.11	[2.01	2.22]	2.98	[2.86	3.10]

Vision Disab.	Est.	95% C.I.		Est.	95% C.I.		Est.	95% C.I.	
Brazil	18.5	[18.4	18.5]	15.5	[15.4	15.5]	21.4	[21.3	21.5]
Costa Rica	4.95	[4.85	5.05]	4.42	[4.30	4.55]	5.44	[5.31	5.58]
Dominican R.	7.83	[7.75	7.91]	5.70	[5.61	5.80]	9.98	[9.86	10.1]
Ecuador	0.96	[0.93	0.98]	1.07	[1.03	1.10]	0.86	[0.82	0.89]
Mexico	0.92	[0.90	0.93]	0.97	[0.95	1.00]	0.86	[0.84	0.89]
Panama	3.74	[3.64	3.83]	3.29	[3.18	3.42]	4.18	[4.04	4.31]
Trinidad & T.	1.07	[0.98	1.17]	0.95	[0.84	1.06]	1.20	[1.07	1.34]
Uruguay	7.11	[6.98	7.25]	5.69	[5.53	5.86]	8.46	[8.27	8.66]

Hearing Disab.	Est.	95% C.I.		Est.	95% C.I.		Est.	95% C.I.	
Brazil	3.57	[3.55	3.59]	3.77	[3.74	3.80]	3.37	[3.35	3.40]
Costa Rica	0.83	[0.80	0.87]	0.91	[0.86	0.97]	0.76	[0.71	0.81]
Dominican R.	1.00	[0.97	1.03]	0.97	[0.93	1.01]	1.03	[0.99	1.07]
Ecuador	0.53	[0.51	0.55]	0.57	[0.55	0.60]	0.48	[0.46	0.51]
Mexico	0.27	[0.26	0.28]	0.31	[0.30	0.32]	0.23	[0.22	0.24]
Panama	0.65	[0.61	0.69]	0.65	[0.60	0.71]	0.65	[0.59	0.70]
Trinidad & T.	0.28	[0.24	0.33]	0.31	[0.25	0.38]	0.26	[0.20	0.32]
Uruguay	1.51	[1.45	1.57]	1.54	[1.45	1.63]	1.48	[1.40	1.57]

Cognitive Disab.	Est.	95% C.I.		Est.	95% C.I.		Est.	95% C.I.	
Brazil	1.34	[1.32	1.35]	1.52	[1.51	1.54]	1.16	[1.14	1.17]
Costa Rica	0.76	[0.72	0.79]	0.86	[0.81	0.92]	0.65	[0.61	0.70]
Dominican R.	2.07	[2.03	2.11]	1.78	[1.72	1.83]	2.36	[2.30	2.43]
Ecuador	0.67	[0.65	0.69]	0.75	[0.72	0.78]	0.59	[0.56	0.62]
Mexico	0.15	[0.14	0.15]	0.16	[0.15	0.17]	0.14	[0.13	0.14]
Panama	1.02	[0.97	1.07]	1.08	[1.01	1.15]	0.96	[0.90	1.03]
Trinidad & T.	0.54	[0.48	0.60]	0.56	[0.47	0.65]	0.52	[0.44	0.61]
Uruguay	1.76	[1.69	1.83]	1.92	[1.82	2.03]	1.61	[1.53	1.70]

Source: authors' estimations based on data provided by Minnesota Population Center (IPUMS International, 2018) from censuses and surveys collected by National Statistics Offices in each country. Estimates for Brazil, Dominican Republic, Ecuador, Mexico, and Panama refer to the year 2010. Estimates for Costa Rica, Trinidad and Tobago, and Uruguay refer to the year 2011.

Table S8.5: Prevalence of Disability by Country and Sex (Ages 56+): Estimates

	Both Sexes			Men			Women		
Motor Disab.	Est.	95% C.I.		Est.	95% C.I.		Est.	95% C.I.	
Brazil	29.2	[29.1	29.3]	22.9	[22.8	23.0]	34.4	[34.3	34.6]
Costa Rica	16.6	[16.2	16.9]	15.1	[14.7	15.5]	17.9	[17.4	18.3]
Dominican R.	22.4	[22.1	22.6]	18.2	[17.9	18.5]	26.4	[26.0	26.7]
Ecuador	8.89	[8.75	9.03]	8.99	[8.79	9.19]	8.80	[8.62	8.99]
Mexico	16.0	[15.8	16.3]	14.5	[14.0	15.0]	17.4	[17.2	17.6]
Panama	14.6	[14.3	15.0]	13.5	[13.1	14.0]	15.7	[15.2	16.1]
Trinidad & T.	6.84	[6.47	7.22]	5.97	[5.49	6.47]	7.63	[7.12	8.18]
Uruguay	21.9	[21.6	22.2]	15.5	[15.1	15.9]	26.6	[26.2	27.1]
Vision Disab.	Est.	95% C.I.		Est.	95% C.I.		Est.	95% C.I.	
Brazil	46.4	[46.3	46.5]	43.4	[43.2	43.5]	48.9	[48.7	49.0]
Costa Rica	18.5	[18.1	18.8]	17.6	[17.2	18.1]	19.2	[18.8	19.7]
Dominican R.	32.2	[31.9	32.4]	28.0	[27.6	28.4]	36.1	[35.7	36.5]
Ecuador	5.18	[5.07	5.29]	5.30	[5.15	5.46]	5.07	[4.92	5.22]
Mexico	6.58	[6.32	6.85]	6.45	[5.92	7.01]	6.69	[6.56	6.83]
Panama	18.7	[18.3	19.0]	17.2	[16.7	17.7]	20.0	[19.5	20.5]
Trinidad & T.	5.48	[5.15	5.83]	4.91	[4.47	5.36]	6.00	[5.55	6.48]
Uruguay	24.6	[24.3	24.9]	20.7	[20.2	21.1]	27.5	[27.1	27.9]
Hearing Disab.	Est.	95% C.I.		Est.	95% C.I.		Est.	95% C.I.	
Brazil	18.9	[18.8	18.9]	20.5	[20.3	20.6]	17.5	[17.4	17.6]
Costa Rica	7.60	[7.37	7.82]	8.29	[7.96	8.62]	6.99	[6.71	7.28]
Dominican R.	8.60	[8.43	8.77]	8.50	[8.27	8.74]	8.69	[8.46	8.93]
Ecuador	3.72	[3.63	3.82]	4.06	[3.93	4.20]	3.40	[3.29	3.53]
Mexico	3.56	[3.48	3.64]	4.03	[3.92	4.13]	3.14	[3.02	3.27]
Panama	5.87	[5.65	6.09]	6.15	[5.84	6.47]	5.60	[5.31	5.90]
Trinidad & T.	1.74	[1.56	1.95]	1.61	[1.36	1.89]	1.86	[1.60	2.14]
Uruguay	12.3	[12.1	12.6]	12.3	[11.9	12.7]	12.3	[12.0	12.7]
Cognitive Disab.	Est.	95% C.I.		Est.	95% C.I.		Est.	95% C.I.	
Brazil	2.44	[2.41	2.48]	2.40	[2.35	2.44]	2.48	[2.44	2.53]
Costa Rica	0.80	[0.73	0.88]	0.87	[0.76	0.99]	0.73	[0.64	0.84]
Dominican R.	11.7	[11.5	11.9]	9.09	[8.85	9.34]	14.2	[13.9	14.5]
Ecuador	1.05	[1.00	1.10]	1.04	[0.97	1.11]	1.06	[1.00	1.13]
Mexico	0.51	[0.48	0.53]	0.44	[0.41	0.47]	0.56	[0.53	0.60]
Panama	3.95	[3.76	4.14]	3.79	[3.54	4.05]	4.10	[3.84	4.36]
Trinidad & T.	1.51	[1.34	1.69]	1.23	[1.01	1.47]	1.76	[1.51	2.04]
Uruguay	4.33	[4.18	4.49]	3.37	[3.17	3.58]	5.05	[4.84	5.27]

Source: authors' estimations based on data provided by Minnesota Population Center (IPUMS International, 2018) from censuses and surveys collected by National Statistics Offices in each country. Estimates for Brazil, Dominican Republic, Ecuador, Mexico, and Panama refer to the year 2010. Estimates for Costa Rica, Trinidad and Tobago, and Uruguay refer to the year 2011.
