# Supplementary material for: Prevalence and correlates of disability in Latin America and the Caribbean: Evidence from 8 national censuses
Source: PLoS One. 2021 Oct 27;16(10):e0258825. doi: 10.1371/journal.pone.0258825 (PMC8550602; doi:10.1371/journal.pone.0258825)
Supplement: S9 Table — (PDF) [file pone.0258825.s009.pdf]

Table S9.1: Prevalence of Disability by Country and Sex (Ages 3 and older): Estimates by Household Head Education Level

	Both Sexes			Men			Women		
Less than Primary	Est.	95% C.I.		Est.	95% C.I.		Est.	95% C.I.	
Brazil	30.7	[30.6	30.8]	27.2	[27.1	27.2]	34.2	[34.1	34.3]
Costa Rica	16.8	[16.5	17.2]	16.5	[16.1	16.9]	17.2	[16.8	17.6]
Dominican R.	16.7	[16.6	16.9]	14.2	[14.0	14.3]	19.5	[19.3	19.7]
Ecuador	8.40	[8.29	8.51]	9.06	[8.91	9.20]	7.75	[7.62	7.89]
Mexico	9.51	[9.38	9.64]	9.58	[9.34	9.82]	9.45	[9.34	9.56]
Panama	12.8	[12.4	13.1]	12.8	[12.4	13.2]	12.7	[12.3	13.2]
Trinidad & T.	7.44	[6.99	7.91]	6.97	[6.42	7.56]	7.91	[7.31	8.56]
Uruguay	34.1	[33.5	34.7]	29.6	[28.9	30.3]	38.3	[37.5	39.0]
Primary Comp.	Est.	95% C.I.		Est.	95% C.I.		Est.	95% C.I.	
Brazil	21.7	[21.6	21.8]	19.1	[19.0	19.2]	24.2	[24.1	24.3]
Costa Rica	9.82	[9.66	9.98]	9.72	[9.52	9.92]	9.91	[9.71	10.1]
Dominican R.	10.8	[10.7	10.9]	9.39	[9.23	9.55]	12.2	[12.0	12.4]
Ecuador	4.88	[4.82	4.95]	5.39	[5.30	5.48]	4.38	[4.30	4.46]
Mexico	4.20	[4.15	4.24]	4.27	[4.21	4.33]	4.13	[4.07	4.19]
Panama	7.85	[7.68	8.01]	7.44	[7.24	7.65]	8.26	[8.04	8.49]
Trinidad & T.	5.22	[4.95	5.49]	5.09	[4.75	5.44]	5.35	[4.99	5.72]
Uruguay	16.0	[15.8	16.2]	13.4	[13.2	13.7]	18.5	[18.2	18.7]
Secondary Comp.	Est.	95% C.I.		Est.	95% C.I.		Est.	95% C.I.	
Brazil	19.0	[18.9	19.1]	16.8	[16.7	16.9]	21.1	[21.0	21.2]
Costa Rica	9.03	[8.75	9.31]	8.58	[8.25	8.93]	9.44	[9.08	9.81]
Dominican R.	9.74	[9.56	9.92]	8.37	[8.16	8.58]	11.0	[10.8	11.2]
Ecuador	3.34	[3.27	3.41]	3.50	[3.40	3.59]	3.19	[3.10	3.29]
Mexico	2.98	[2.90	3.06]	2.93	[2.82	3.04]	3.03	[2.93	3.13]
Panama	6.11	[5.92	6.31]	5.65	[5.41	5.90]	6.55	[6.29	6.82]
Trinidad & T.	2.95	[2.78	3.13]	2.92	[2.70	3.15]	2.97	[2.75	3.21]
Uruguay	12.1	[11.8	12.3]	9.74	[9.41	10.1]	14.1	[13.7	14.4]
University Comp.	Est.	95% C.I.		Est.	95% C.I.		Est.	95% C.I.	
Brazil	19.1	[19.0	19.3]	17.5	[17.3	17.7]	20.5	[20.3	20.7]
Costa Rica	7.67	[7.41	7.93]	7.39	[7.07	7.72]	7.92	[7.60	8.25]
Dominican R.	11.4	[11.1	11.7]	10.3	[9.97	10.6]	12.4	[12.0	12.7]
Ecuador	2.94	[2.83	3.06]	3.02	[2.87	3.18]	2.87	[2.73	3.02]
Mexico	2.61	[2.52	2.69]	2.62	[2.49	2.75]	2.60	[2.49	2.70]
Panama	6.65	[6.37	6.95]	6.01	[5.66	6.38]	7.21	[6.84	7.60]
Trinidad & T.	3.17	[2.53	3.92]	2.82	[2.14	3.70]	3.50	[2.64	4.48]
Uruguay	8.52	[8.08	8.98]	7.62	[7.06	8.20]	9.31	[8.74	9.92]

Source: authors' estimations based on data provided by Minnesota Population Center (IPUMS International, 2018) from censuses and surveys collected by National Statistics Offices in each country. Estimates for Brazil, Dominican Republic, Ecuador, Mexico, and Panama refer to the year 2010. Estimates for Costa Rica, Trinidad and Tobago, and Uruguay refer to the year 2011.

Table S9.2: Prevalence of Disability by Country and Sex (Ages 3-5): Estimates by Household Head Education Level

	Both Sexes			Men			Women		
Less than Primary	Est.	95% C.I.		Est.	95% C.I.		Est.	95% C.I.	
Brazil	4.30	[4.20	4.41]	4.64	[4.48	4.79]	3.96	[3.82	4.11]
Costa Rica	2.84	[2.35	3.42]	3.23	[2.53	4.08]	2.42	[1.75	3.20]
Dominican R.	2.33	[2.11	2.56]	2.52	[2.21	2.87]	2.13	[1.83	2.45]
Ecuador	2.00	[1.81	2.21]	2.29	[2.00	2.61]	1.71	[1.45	1.99]
Mexico	1.40	[1.30	1.51]	1.65	[1.48	1.83]	1.14	[1.03	1.27]
Panama	1.30	[0.97	1.74]	1.39	[0.88	2.06]	1.22	[0.77	1.78]
Trinidad & T.	0.34	[0.042	1.24]	0.68	[0.083	2.44]	0	[0	0]
Uruguay	2.98	[1.97	4.30]	2.85	[1.53	4.62]	3.10	[1.70	4.93]

Primary Comp.	Est.	95% C.I.		Est.	95% C.I.		Est.	95% C.I.	
Brazil	3.98	[3.85	4.11]	4.25	[4.07	4.44]	3.69	[3.51	3.87]
Costa Rica	2.22	[1.94	2.52]	2.53	[2.14	3.00]	1.89	[1.53	2.30]
Dominican R.	1.83	[1.64	2.03]	2.22	[1.94	2.55]	1.41	[1.17	1.66]
Ecuador	1.83	[1.70	1.97]	2.05	[1.86	2.25]	1.60	[1.43	1.79]
Mexico	1.30	[1.23	1.38]	1.47	[1.37	1.59]	1.13	[1.04	1.22]
Panama	1.19	[0.97	1.44]	1.22	[0.92	1.58]	1.15	[0.86	1.52]
Trinidad & T.	0.93	[0.49	1.58]	1.57	[0.78	2.78]	0.29	[0.035	1.03]
Uruguay	2.03	[1.74	2.36]	2.28	[1.86	2.77]	1.77	[1.38	2.22]

Secondary Comp.	Est.	95% C.I.		Est.	95% C.I.		Est.	95% C.I.	
Brazil	3.21	[3.08	3.35]	3.22	[3.04	3.41]	3.21	[3.01	3.41]
Costa Rica	1.85	[1.39	2.41]	2.38	[1.65	3.30]	1.32	[0.80	2.06]
Dominican R.	1.45	[1.23	1.71]	1.69	[1.37	2.09]	1.21	[0.93	1.54]
Ecuador	1.45	[1.29	1.62]	1.43	[1.22	1.67]	1.48	[1.25	1.73]
Mexico	1.21	[1.06	1.36]	1.42	[1.20	1.66]	0.99	[0.81	1.18]
Panama	0.80	[0.56	1.11]	0.80	[0.48	1.27]	0.80	[0.46	1.27]
Trinidad & T.	0.78	[0.46	1.23]	1.09	[0.58	1.87]	0.44	[0.14	1.03]
Uruguay	1.52	[1.06	2.09]	1.34	[0.74	2.16]	1.71	[1.06	2.61]

University Comp.	Est.	95% C.I.		Est.	95% C.I.		Est.	95% C.I.	
Brazil	2.39	[2.16	2.63]	2.61	[2.29	2.97]	2.16	[1.86	2.49]
Costa Rica	1.49	[1.07	2.01]	1.19	[0.71	1.87]	1.82	[1.18	2.68]
Dominican R.	1.58	[1.22	1.99]	2.00	[1.46	2.68]	1.13	[0.71	1.70]
Ecuador	1.03	[0.78	1.32]	1.18	[0.82	1.63]	0.86	[0.55	1.28]
Mexico	0.95	[0.79	1.14]	1.21	[0.94	1.52]	0.69	[0.52	0.90]
Panama	1.00	[0.58	1.58]	1.10	[0.55	1.96]	0.90	[0.39	1.76]
Trinidad & T.	0.56	[0.014	3.09]	.	[.	.]	.	[.	.]
Uruguay	1.61	[0.79	2.79]	1.75	[0.70	3.55]	1.49	[0.54	3.18]

Source: authors' estimations based on data provided by Minnesota Population Center (IPUMS International, 2018) from censuses and surveys collected by National Statistics Offices in each country. Estimates for Brazil, Dominican Republic, Ecuador, Mexico, and Panama refer to the year 2010. Estimates for Costa Rica, Trinidad and Tobago, and Uruguay refer to the year 2011.

Table S9.3: Prevalence of Disability by Country and Sex (Ages 6-17): Estimates by Household Head Education Level

	Both Sexes			Men			Women		
Less than Primary	Est.	95% C.I.		Est.	95% C.I.		Est.	95% C.I.	
Brazil	11.2	[11.1	11.3]	10.4	[10.3	10.5]	12.0	[11.9	12.1]
Costa Rica	5.55	[5.19	5.91]	6.09	[5.60	6.61]	4.99	[4.53	5.47]
Dominican R.	4.43	[4.29	4.58]	4.39	[4.19	4.58]	4.48	[4.29	4.69]
Ecuador	3.21	[3.09	3.34]	3.53	[3.36	3.71]	2.88	[2.72	3.04]
Mexico	2.27	[2.21	2.34]	2.55	[2.46	2.63]	2.00	[1.91	2.08]
Panama	3.12	[2.83	3.43]	3.48	[3.07	3.92]	2.75	[2.38	3.14]
Trinidad & T.	1.81	[1.32	2.46]	1.41	[0.83	2.20]	2.23	[1.47	3.23]
Uruguay	12.1	[11.1	13.1]	12.9	[11.6	14.3]	11.2	[9.99	12.5]
Primary Comp.	Est.	95% C.I.		Est.	95% C.I.		Est.	95% C.I.	
Brazil	10.7	[10.6	10.8]	9.77	[9.63	9.91]	11.6	[11.4	11.7]
Costa Rica	4.49	[4.29	4.69]	4.93	[4.64	5.22]	4.04	[3.79	4.31]
Dominican R.	4.09	[3.95	4.24]	3.89	[3.70	4.08]	4.29	[4.09	4.50]
Ecuador	2.69	[2.61	2.77]	3.03	[2.91	3.15]	2.34	[2.23	2.45]
Mexico	2.01	[1.96	2.07]	2.28	[2.19	2.37]	1.74	[1.68	1.80]
Panama	2.68	[2.50	2.87]	2.98	[2.73	3.25]	2.37	[2.14	2.61]
Trinidad & T.	1.60	[1.27	1.97]	1.86	[1.37	2.44]	1.32	[0.90	1.84]
Uruguay	7.92	[7.63	8.22]	8.37	[7.98	8.79]	7.45	[7.07	7.86]
Secondary Comp.	Est.	95% C.I.		Est.	95% C.I.		Est.	95% C.I.	
Brazil	9.37	[9.25	9.50]	8.63	[8.47	8.80]	10.1	[9.94	10.3]
Costa Rica	4.15	[3.78	4.54]	4.06	[3.56	4.61]	4.23	[3.72	4.80]
Dominican R.	4.08	[3.87	4.30]	3.73	[3.46	4.02]	4.42	[4.12	4.73]
Ecuador	2.19	[2.08	2.30]	2.39	[2.24	2.54]	1.98	[1.85	2.13]
Mexico	1.58	[1.49	1.67]	1.86	[1.73	1.99]	1.29	[1.18	1.42]
Panama	2.25	[2.03	2.49]	2.57	[2.24	2.93]	1.92	[1.63	2.23]
Trinidad & T.	1.13	[0.91	1.38]	1.15	[0.85	1.50]	1.11	[0.80	1.47]
Uruguay	5.20	[4.76	5.67]	5.22	[4.64	5.87]	5.17	[4.57	5.82]
University Comp.	Est.	95% C.I.		Est.	95% C.I.		Est.	95% C.I.	
Brazil	8.35	[8.13	8.57]	8.10	[7.81	8.40]	8.60	[8.30	8.91]
Costa Rica	3.35	[3.02	3.71]	3.34	[2.89	3.85]	3.37	[2.92	3.89]
Dominican R.	4.02	[3.72	4.33]	3.51	[3.13	3.92]	4.51	[4.09	4.97]
Ecuador	1.55	[1.39	1.73]	1.75	[1.52	2.01]	1.35	[1.14	1.58]
Mexico	1.39	[1.28	1.51]	1.74	[1.57	1.93]	1.02	[0.90	1.15]
Panama	2.32	[1.97	2.70]	2.48	[2.00	3.02]	2.16	[1.71	2.71]
Trinidad & T.	1.70	[0.75	3.09]	2.11	[0.63	4.46]	1.27	[0.35	3.24]
Uruguay	3.17	[2.56	3.84]	3.26	[2.45	4.30]	3.07	[2.25	4.04]

Source: authors' estimations based on data provided by Minnesota Population Center (IPUMS International, 2018) from censuses and surveys collected by National Statistics Offices in each country. Estimates for Brazil, Dominican Republic, Ecuador, Mexico, and Panama refer to the year 2010. Estimates for Costa Rica, Trinidad and Tobago, and Uruguay refer to the year 2011.

Table S9.4: Prevalence of Disability by Country and Sex (Ages 18-55): Estimates by Household Head Education Level

	Both Sexes			Men			Women		
Less than Primary	Est.	95% C.I.		Est.	95% C.I.		Est.	95% C.I.	
Brazil	27.3	[27.2	27.4]	23.7	[23.6	23.8]	31.0	[30.9	31.1]
Costa Rica	11.4	[11.1	11.7]	11.2	[10.8	11.7]	11.6	[11.2	12.0]
Dominican R.	12.8	[12.7	13.0]	10.7	[10.5	10.9]	15.4	[15.1	15.6]
Ecuador	6.55	[6.41	6.68]	7.39	[7.20	7.58]	5.72	[5.55	5.89]
Mexico	5.34	[5.26	5.41]	5.81	[5.71	5.92]	4.91	[4.82	5.00]
Panama	8.48	[8.12	8.86]	8.00	[7.54	8.47]	9.04	[8.53	9.57]
Trinidad & T.	4.76	[4.29	5.26]	5.24	[4.61	5.92]	4.22	[3.62	4.90]
Uruguay	20.0	[19.3	20.8]	18.9	[17.9	19.8]	21.4	[20.3	22.4]
Primary Comp.	Est.	95% C.I.		Est.	95% C.I.		Est.	95% C.I.	
Brazil	22.3	[22.2	22.4]	19.5	[19.4	19.6]	25.0	[24.9	25.2]
Costa Rica	8.39	[8.21	8.57]	8.44	[8.20	8.68]	8.35	[8.12	8.58]
Dominican R.	10.8	[10.6	10.9]	8.95	[8.76	9.15]	12.6	[12.4	12.8]
Ecuador	4.16	[4.08	4.23]	4.86	[4.75	4.97]	3.48	[3.38	3.57]
Mexico	3.17	[3.13	3.22]	3.53	[3.46	3.60]	2.84	[2.79	2.90]
Panama	6.06	[5.87	6.25]	5.88	[5.65	6.13]	6.24	[5.99	6.50]
Trinidad & T.	3.74	[3.45	4.04]	3.89	[3.50	4.29]	3.58	[3.20	4.00]
Uruguay	11.4	[11.1	11.6]	9.67	[9.40	9.95]	13.0	[12.7	13.3]
Secondary Comp.	Est.	95% C.I.		Est.	95% C.I.		Est.	95% C.I.	
Brazil	18.8	[18.7	18.9]	16.7	[16.6	16.9]	20.6	[20.5	20.7]
Costa Rica	7.19	[6.89	7.50]	7.03	[6.65	7.44]	7.33	[6.94	7.73]
Dominican R.	10.0	[9.82	10.2]	8.55	[8.29	8.82]	11.3	[11.0	11.6]
Ecuador	2.81	[2.73	2.89]	3.19	[3.08	3.31]	2.44	[2.35	2.55]
Mexico	2.16	[2.08	2.25]	2.38	[2.25	2.51]	1.97	[1.87	2.07]
Panama	4.94	[4.73	5.16]	4.77	[4.49	5.06]	5.10	[4.82	5.40]
Trinidad & T.	2.27	[2.09	2.46]	2.43	[2.18	2.70]	2.12	[1.88	2.37]
Uruguay	7.94	[7.65	8.23]	7.08	[6.71	7.46]	8.69	[8.31	9.09]
University Comp.	Est.	95% C.I.		Est.	95% C.I.		Est.	95% C.I.	
Brazil	17.3	[17.2	17.5]	16.2	[16.0	16.4]	18.3	[18.1	18.5]
Costa Rica	6.86	[6.58	7.15]	6.93	[6.55	7.32]	6.80	[6.45	7.16]
Dominican R.	11.5	[11.2	11.8]	10.6	[10.2	11.0]	12.2	[11.8	12.6]
Ecuador	2.21	[2.09	2.33]	2.54	[2.36	2.72]	1.92	[1.78	2.07]
Mexico	1.68	[1.60	1.76]	1.84	[1.73	1.95]	1.52	[1.42	1.63]
Panama	5.03	[4.72	5.34]	4.74	[4.34	5.17]	5.26	[4.87	5.68]
Trinidad & T.	2.96	[2.20	3.90]	2.17	[1.40	3.23]	3.64	[2.63	4.99]
Uruguay	5.61	[5.16	6.08]	5.27	[4.68	5.91]	5.89	[5.32	6.53]

Source: authors' estimations based on data provided by Minnesota Population Center (IPUMS International, 2018) from censuses and surveys collected by National Statistics Offices in each country. Estimates for Brazil, Dominican Republic, Ecuador, Mexico, and Panama refer to the year 2010. Estimates for Costa Rica, Trinidad and Tobago, and Uruguay refer to the year 2011.

Table S9.5: Prevalence of Disability by Country and Sex (Ages 56+): Estimates by Household Head Education Level

	Both Sexes			Men			Women		
Less than Primary	Est.	95% C.I.		Est.	95% C.I.		Est.	95% C.I.	
Brazil	66.3	[66.2	66.4]	62.9	[62.7	63.0]	69.2	[69.0	69.3]
Costa Rica	40.5	[39.8	41.2]	40.6	[39.6	41.6]	40.4	[39.5	41.3]
Dominican R.	46.9	[46.5	47.3]	41.5	[41.0	42.1]	51.9	[51.3	52.4]
Ecuador	20.5	[20.2	20.9]	22.2	[21.7	22.6]	19.1	[18.7	19.5]
Mexico	28.0	[27.6	28.5]	27.8	[27.0	28.7]	28.2	[27.8	28.6]
Panama	35.8	[35.0	36.7]	35.7	[34.6	36.8]	36.0	[34.8	37.2]
Trinidad & T.	17.6	[16.3	18.8]	16.1	[14.5	17.9]	18.7	[17.1	20.3]
Uruguay	53.8	[53.1	54.6]	47.9	[46.8	49.0]	58.4	[57.4	59.3]
Primary Comp.	Est.	95% C.I.		Est.	95% C.I.		Est.	95% C.I.	
Brazil	57.3	[57.0	57.6]	53.3	[52.9	53.7]	60.7	[60.3	61.0]
Costa Rica	32.1	[31.4	32.8]	30.9	[30.0	31.7]	33.3	[32.4	34.2]
Dominican R.	41.3	[40.7	42.0]	36.6	[35.8	37.5]	46.2	[45.3	47.1]
Ecuador	16.8	[16.4	17.1]	17.0	[16.6	17.4]	16.5	[16.1	16.9]
Mexico	19.4	[19.1	19.7]	17.7	[17.4	18.1]	20.9	[20.5	21.3]
Panama	28.8	[28.1	29.5]	26.4	[25.5	27.3]	31.2	[30.2	32.1]
Trinidad & T.	12.5	[11.8	13.4]	11.6	[10.6	12.7]	13.5	[12.4	14.6]
Uruguay	40.1	[39.6	40.7]	34.2	[33.5	35.0]	44.6	[43.9	45.3]
Secondary Comp.	Est.	95% C.I.		Est.	95% C.I.		Est.	95% C.I.	
Brazil	50.2	[49.8	50.5]	45.6	[45.2	46.1]	53.6	[53.2	54.0]
Costa Rica	28.6	[27.5	29.8]	27.6	[26.1	29.1]	29.5	[28.0	30.9]
Dominican R.	38.2	[37.1	39.2]	33.5	[32.1	34.9]	42.3	[40.9	43.7]
Ecuador	13.6	[13.2	14.1]	12.8	[12.2	13.5]	14.3	[13.7	14.9]
Mexico	15.3	[14.8	16.0]	13.1	[12.3	13.9]	17.1	[16.4	17.9]
Panama	24.8	[23.7	25.9]	22.8	[21.4	24.2]	26.3	[25.0	27.7]
Trinidad & T.	9.65	[8.86	10.5]	8.81	[7.80	9.90]	10.4	[9.36	11.6]
Uruguay	31.6	[30.7	32.4]	25.4	[24.3	26.6]	35.7	[34.6	36.7]
University Comp.	Est.	95% C.I.		Est.	95% C.I.		Est.	95% C.I.	
Brazil	41.4	[40.9	41.8]	38.0	[37.5	38.6]	43.9	[43.4	44.4]
Costa Rica	21.0	[20.0	22.0]	19.3	[18.0	20.6]	22.5	[21.2	23.9]
Dominican R.	34.8	[33.6	36.0]	30.5	[29.0	32.0]	38.9	[37.3	40.5]
Ecuador	10.0	[9.51	10.6]	8.48	[7.83	9.19]	11.6	[10.8	12.4]
Mexico	11.5	[10.9	12.0]	9.39	[8.56	10.3]	13.7	[13.0	14.4]
Panama	21.5	[20.3	22.7]	19.5	[17.9	21.1]	23.0	[21.6	24.6]
Trinidad & T.	5.45	[4.08	7.22]	5.57	[3.66	8.16]	5.33	[3.34	7.90]
Uruguay	23.1	[21.7	24.6]	20.3	[18.4	22.3]	25.3	[23.5	27.2]

Source: authors' estimations based on data provided by Minnesota Population Center (IPUMS International, 2018) from censuses and surveys collected by National Statistics Offices in each country. Estimates for Brazil, Dominican Republic, Ecuador, Mexico, and Panama refer to the year 2010. Estimates for Costa Rica, Trinidad and Tobago, and Uruguay refer to the year 2011.
