# Supplementary material for: Prevalence and correlates of disability in Latin America and the Caribbean: Evidence from 8 national censuses
Source: PLoS One. 2021 Oct 27;16(10):e0258825. doi: 10.1371/journal.pone.0258825 (PMC8550602; doi:10.1371/journal.pone.0258825)
Supplement: S10 Table — (PDF) [file pone.0258825.s010.pdf]

Table S10.1: **Estimates for School Attendance and Completion Rates and Gaps (Female)**

	Attendance: 6-11			Attendance: 12-17			Completion: 25-34		
	(1) No disab.	(2) Any disab.	(3) Gap	(4) No disab.	(5) Any disab.	(6) Gap	(7) No disab.	(8) Any disab.	(9) Gap
Brazil	0.975	0.965	$0.010^{\ddagger}$	0.898	0.891	$0.008^{\ddagger}$	0.545	0.468	$0.077^{\ddagger}$
			(0.001)			(0.002)			(0.002)
Costa Rica	0.941	0.969	$-0.028^{\ddagger}$	0.845	0.841	0.004	0.461	0.423	$0.039^{\ddagger}$
			(0.006)			(0.011)			(0.012)
Dominican R.	0.963	0.858	$0.105^{\ddagger}$	0.881	0.808	$0.073^{\ddagger}$	0.508	0.453	$0.056^{\ddagger}$
			(0.008)			(0.007)			(0.007)
Ecuador	0.971	0.840	$0.131^{\ddagger}$	0.832	0.666	$0.166^{\ddagger}$	0.494	0.289	$0.205^{\ddagger}$
			(0.008)			(0.011)			(0.009)
Mexico	0.971	0.827	$0.144^{\ddagger}$	0.799	0.635	$0.164^{\ddagger}$	0.385	0.197	$0.188^{\ddagger}$
			(0.007)			(0.009)			(0.006)
Panama	0.975	0.932	$0.043^{\ddagger}$	0.884	0.848	$0.035^{\dagger}$	0.584	0.490	$0.094^{\ddagger}$
			(0.012)			(0.017)			(0.019)
Trinidad & T	0.991	0.840	$0.151^{\ddagger}$	0.928	0.758	$0.170^{\ddagger}$	0.816	0.567	$0.248^{\ddagger}$
			(0.052)			(0.054)			(0.039)
Uruguay	0.993	0.975	$0.018^{\ddagger}$	0.870	0.816	$0.054^{\ddagger}$	0.440	0.294	$0.146^{\ddagger}$
			(0.005)			(0.012)			(0.012)
Average	0.972	0.901	0.072	0.867	0.783	0.084	0.529	0.398	0.132

Source: authors' estimations based on data provided by Minnesota Population Center (IPUMS International, 2018) from censuses collected by National Statistics Offices in each country. Estimates for Brazil, Dominican Republic, Ecuador, Mexico, and Panama refer to year 2010. Estimates for Costa Rica, Trinidad and Tobago, and Uruguay refer to year 2011. Symbols in columns (3), (6) and (9) denote statistical significance: * 0.10, † 0.05, and ‡ 0.01.

Table S10.2: **Estimates for School Attendance and Completion Rates and Gaps (Male).**

	Attendance: 6-11			Attendance: 12-17			Completion: 25-34		
	(1) No disab.	(2) Any disab.	(3) Gap	(4) No disab.	(5) Any disab.	(6) Gap	(7) No disab.	(8) Any disab.	(9) Gap
Brazil	0.972	0.949	0.023‡	0.896	0.873	0.023‡	0.465	0.405	0.060‡
			(0.001)			(0.002)			(0.002)
Costa Rica	0.944	0.944	-0.000	0.830	0.783	0.047‡	0.409	0.343	0.066‡
			(0.007)			(0.013)			(0.011)
Dominican R.	0.950	0.801	0.149‡	0.865	0.704	0.161‡	0.377	0.305	0.072‡
			(0.009)			(0.009)			(0.007)
Ecuador	0.968	0.816	0.153‡	0.837	0.642	0.195‡	0.480	0.276	0.204‡
			(0.007)			(0.009)			(0.007)
Mexico	0.968	0.826	0.142‡	0.790	0.605	0.184‡	0.386	0.182	0.204‡
			(0.006)			(0.008)			(0.008)
Panama	0.973	0.948	0.025‡	0.882	0.821	0.060‡	0.491	0.378	0.113‡
			(0.009)			(0.016)			(0.017)
Trinidad & T	0.988	0.800	0.188‡	0.901	0.681	0.220‡	0.759	0.516	0.243‡
			(0.054)			(0.055)			(0.034)
Uruguay	0.992	0.976	0.015‡	0.812	0.740	0.072‡	0.341	0.219	0.122‡
			(0.004)			(0.013)			(0.011)
Average	0.969	0.883	0.087	0.852	0.731	0.121	0.464	0.328	0.135

Source: authors' estimations based on data provided by Minnesota Population Center (IPUMS International, 2018) from censuses collected by National Statistics Offices in each country. Estimates for Brazil, Dominican Republic, Ecuador, Mexico, and Panama refer to year 2010. Estimates for Costa Rica, Trinidad and Tobago, and Uruguay refer to year 2011. Symbols in columns (3), (6) and (9) denote statistical significance: * 0.10, † 0.05, and ‡ 0.01.
