# Supplementary material for: Prevalence and correlates of disability in Latin America and the Caribbean: Evidence from 8 national censuses
Source: PLoS One. 2021 Oct 27;16(10):e0258825. doi: 10.1371/journal.pone.0258825 (PMC8550602; doi:10.1371/journal.pone.0258825)
Supplement: S13 Table — (PDF) [file pone.0258825.s013.pdf]

Table S13.1: **Estimates for Employment Rates and Disability Gaps (Vision Disabilities).**

	Employment Rate: 25-34			Employment Rate: 35-54		
	(1) No disab.	(2) Any disab.	(3) Gap	(4) No disab.	(5) Any disab.	(6) Gap
Brazil	0.724	0.671	0.053‡	0.740	0.636	0.104‡
			(0.001)			(0.001)
Costa Rica	0.639	0.640	-0.001	0.624	0.539	0.085‡
			(0.011)			(0.006)
Dominican R.	0.585	0.532	0.053‡	0.589	0.494	0.095‡
			(0.007)			(0.003)
Ecuador	0.698	0.607	0.091‡	0.718	0.610	0.108‡
			(0.014)			(0.008)
Mexico	0.667	0.618	0.049‡	0.680	0.579	0.100‡
			(0.010)			(0.006)
Panama	0.694	0.713	-0.019	0.720	0.647	0.073‡
			(0.017)			(0.007)
Trinidad & T	0.778	0.673	0.105†	0.778	0.544	0.234‡
			(0.048)			(0.024)
Uruguay	0.829	0.731	0.098‡	0.848	0.713	0.136‡
			(0.010)			(0.005)
Average	0.702	0.648	0.054	0.712	0.595	0.117

Source: authors' estimations based on data provided by Minnesota Population Center (IPUMS International, 2018) from censuses collected by National Statistics Offices in each country. Estimates for Brazil, Dominican Republic, Ecuador, Mexico, and Panama refer to year 2010. Estimates for Costa Rica, Trinidad and Tobago, and Uruguay refer to year 2011. Symbols in columns (3), (6) and (9) denote statistical significance: * 0.10, † 0.05, and ‡ 0.01.

Table S13.2: **Estimates for Employment Rates and Disability Gaps (Hearing Disabilities).**

	Employment Rate: 25-34			Employment Rate: 35-54		
	(1) No disab.	(2) Any disab.	(3) Gap	(4) No disab.	(5) Any disab.	(6) Gap
Brazil	0.724	0.652	0.072‡	0.740	0.621	0.119‡
			(0.003)			(0.002)
Costa Rica	0.639	0.488	0.151‡	0.624	0.501	0.123‡
			(0.027)			(0.014)
Dominican R.	0.585	0.451	0.133‡	0.589	0.414	0.175‡
			(0.016)			(0.009)
Ecuador	0.698	0.531	0.166‡	0.718	0.552	0.166‡
			(0.019)			(0.011)
Mexico	0.667	0.529	0.137‡	0.680	0.596	0.084‡
			(0.016)			(0.009)
Panama	0.694	0.542	0.152‡	0.720	0.542	0.178‡
			(0.034)			(0.018)
Trinidad & T	0.778	0.341	0.437‡	0.778	0.327	0.451‡
			(0.072)			(0.046)
Uruguay	0.829	0.674	0.155‡	0.848	0.717	0.131‡
			(0.024)			(0.011)
Average	0.702	0.526	0.175	0.712	0.534	0.178

Source: authors' estimations based on data provided by Minnesota Population Center (IPUMS International, 2018) from censuses collected by National Statistics Offices in each country. Estimates for Brazil, Dominican Republic, Ecuador, Mexico, and Panama refer to year 2010. Estimates for Costa Rica, Trinidad and Tobago, and Uruguay refer to year 2011. Symbols in columns (3), (6) and (9) denote statistical significance: * 0.10, † 0.05, and ‡ 0.01.

Table S13.3: **Estimates for Employment Rates and Disability Gaps (Motor Disabilities).**

	Employment Rate: 25-34			Employment Rate: 35-54		
	(1) No disab.	(2) Any disab.	(3) Gap	(4) No disab.	(5) Any disab.	(6) Gap
Brazil	0.724	0.522	0.202‡	0.740	0.493	0.247‡
			(0.003)			(0.001)
Costa Rica	0.639	0.455	0.185‡	0.624	0.412	0.212‡
			(0.017)			(0.008)
Dominican R.	0.585	0.456	0.129‡	0.589	0.404	0.185‡
			(0.009)			(0.005)
Ecuador	0.698	0.553	0.144‡	0.718	0.529	0.189‡
			(0.009)			(0.005)
Mexico	0.667	0.489	0.178‡	0.680	0.486	0.193‡
			(0.009)			(0.004)
Panama	0.694	0.436	0.258‡	0.720	0.489	0.231‡
			(0.024)			(0.011)
Trinidad & T	0.778	0.315	0.463‡	0.778	0.258	0.521‡
			(0.041)			(0.019)
Uruguay	0.829	0.527	0.302‡	0.848	0.503	0.346‡
			(0.021)			(0.009)
Average	0.702	0.469	0.233	0.712	0.447	0.265

Source: authors' estimations based on data provided by Minnesota Population Center (IPUMS International, 2018) from censuses collected by National Statistics Offices in each country. Estimates for Brazil, Dominican Republic, Ecuador, Mexico, and Panama refer to year 2010. Estimates for Costa Rica, Trinidad and Tobago, and Uruguay refer to year 2011. Symbols in columns (3), (6) and (9) denote statistical significance: * 0.10, † 0.05, and ‡ 0.01.

Table S13.4: **Estimates for Employment Rates and Disability Gaps (Cognitive Disabilities).**

	Employment Rate: 25-34			Employment Rate: 35-54		
	(1) No disab.	(2) Any disab.	(3) Gap	(4) No disab.	(5) Any disab.	(6) Gap
Brazil	0.724	0.200	0.524‡	0.740	0.217	0.523‡
			(0.004)			(0.003)
Costa Rica	0.639	0.154	0.485‡	0.624	0.101	0.523‡
			(0.016)			(0.011)
Dominican R.	0.585	0.352	0.233‡	0.589	0.331	0.258‡
			(0.010)			(0.006)
Ecuador	0.698	0.267	0.431‡	0.718	0.280	0.438‡
			(0.012)			(0.010)
Mexico	0.667	0.208	0.458‡	0.680	0.228	0.452‡
			(0.021)			(0.014)
Panama	0.694	0.246	0.447‡	0.720	0.299	0.421‡
			(0.021)			(0.015)
Trinidad & T	0.778	0.227	0.551‡	0.778	0.209	0.569‡
			(0.045)			(0.031)
Uruguay	0.829	0.351	0.478‡	0.848	0.357	0.492‡
			(0.017)			(0.013)
Average	0.702	0.251	0.451	0.712	0.253	0.459

Source: authors' estimations based on data provided by Minnesota Population Center (IPUMS International, 2018) from censuses collected by National Statistics Offices in each country. Estimates for Brazil, Dominican Republic, Ecuador, Mexico, and Panama refer to year 2010. Estimates for Costa Rica, Trinidad and Tobago, and Uruguay refer to year 2011. Symbols in columns (3), (6) and (9) denote statistical significance: * 0.10, † 0.05, and ‡ 0.01.
