# Supplementary material for: Prevalence and correlates of disability in Latin America and the Caribbean: Evidence from 8 national censuses
Source: PLoS One. 2021 Oct 27;16(10):e0258825. doi: 10.1371/journal.pone.0258825 (PMC8550602; doi:10.1371/journal.pone.0258825)
Supplement: S14 Table — (PDF) [file pone.0258825.s014.pdf]

Table S14: Estimates for Disability Prevalence using Dominican Republic 2010 Prevalence Rates

	2020 Population Estimates						2050 Population Estimates					
	# Persons (000s)			Rate (%)			# Persons (000s)			Rate (%)		
	All	Female	Male	All	Female	Male	All	Female	Male	All	Female	Male
Argentina	7,202	4,361	2,841	17.4	20.5	14.1	10,955	6,502	4,453	21.3	24.8	17.7
Bahamas	59.9	35.9	24	16.4	19	13.5	97	58.1	38.9	22.1	25.8	18.3
Belize	46.1	26.2	20	12.9	14.5	11.2	103	60.5	42.1	19.2	22.2	16.1
Bolivia	1,477	847	630	14.1	16.2	12	2,742	1,590	1,152	18.6	21.5	15.8
Brazil	33,674	20,056	13,619	17	19.8	14	54,221	32,091	22,130	24.8	28.7	20.8
Barbados	59.4	35.4	23.9	21.8	25.2	18.2	72.2	41.5	30.7	27.3	30.9	23.6
Chile	3,351	1,987	1,364	18.7	21.8	15.4	5,049	2,894	2,154	26.1	29.6	22.5
Colombia	7,703	4,576	3,127	16.3	19	13.5	12,814	7,494	5,320	24.1	27.7	20.3
Costa Rica	824	476	348	17.4	20	14.7	1,405	804	601	25.5	29	22
Dominican R.	1,469	846	622	14.9	17.1	12.7	2,491	1,457	1,034	20.7	24	17.4
Ecuador	2,370	1,367	1,003	14.8	17.1	12.6	4,505	2,586	1,919	20.6	23.6	17.6
El Salvador	909	566	342	15.4	17.9	12.5	1,410	891	518	21.5	25.5	17
Guatemala	1,910	1,132	778	12.1	14	10	4,392	2,587	1,806	17.6	20.5	14.7
Guyana	105	60.7	44.7	14.8	17.1	12.5	152	88	64.3	19.8	23	16.6
Honduras	1,104	642	462	12.4	14.4	10.4	2,463	1,425	1,038	19	22	16.1
Haiti	1,265	741	524	12.5	14.4	10.5	2,350	1,384	966	17.1	19.9	14.2
Jamaica	445	257	189	16.3	18.6	14	633	372	261	22.6	26	19.1
Mexico	18,028	10,697	7,331	15.3	17.7	12.8	31,427	18,684	12,743	21.5	25	17.8
Nicaragua	800	479	321	13.4	15.8	11	1,605	955	651	20.1	23.5	16.5
Panama	622	358	264	15.8	18.2	13.5	1,172	672	500	21.4	24.4	18.4
Paraguay	879	494	385	13.7	15.6	11.8	1,605	917	687	18.9	21.7	16.1
Peru	4,805	2,747	2,058	15.9	18.1	13.8	8,426	4,852	3,574	22.2	25.2	19
Suriname	81.5	47.8	33.7	15.3	17.9	12.6	125	74.3	51.1	19.7	23.1	16.2
Trinidad & T.	241	141	99.7	18.4	21.3	15.4	314	186	128	24.6	28.3	20.6
Uruguay	652	402	250	20.1	23.9	16	829	494	335	24.1	28.1	19.9
Venezuela	4,071	2,407	1,664	15.6	18.1	13	7,109	4,245	2,865	20.5	23.8	16.9
Other Carib.	2,665	1,562	1,102	21.8	25.2	18.3	3,197	1,837	1,360	28.5	32.4	24.6
Total LAC	96,818	57,348	39,470	16.2	18.8	13.4	161,664	95,241	66,423	22.6	26.1	18.9

Source: authors' estimations based on data provided by Minnesota Population Center (IPUMS International, 2018) from censuses and surveys collected by National Statistics Offices in each country, and population projections by CEPAL (2019). The "Other Carib." category includes Antigua and Barbuda, Aruba, Cuba, Curacao, Granada, Guadeloupe, Martinica, St. Vincent and the Granadines, and St. Lucia. Estimates consider individuals aged 5 years and older.
