# Supplementary material for: Prevalence and correlates of disability in Latin America and the Caribbean: Evidence from 8 national censuses
Source: PLoS One. 2021 Oct 27;16(10):e0258825. doi: 10.1371/journal.pone.0258825 (PMC8550602; doi:10.1371/journal.pone.0258825)
Supplement: S1 Appendix — (PDF) [file pone.0258825.s016.pdf]

A Appendix: Estimation Method

This appendix describes the methodology used to compute the prevalence of disability using information from representative samples by IPUMS-International (Minnesota Population Center, 2018).[1]

Disability Rates: Estimates, Standard Errors, and Confidence Intervals.

We estimate several population statistics using data from a sample of the population obtained from the last available national census provided by IPUMS-International. To perform these estimations accurately and assess their precision and reliability, it is important to consider the procedure used to select the sample from the population, known as *sampling method*.

S2 Table (supporting information) presents the main characteristics of the sampling method for the IPUMS-International samples used in our analysis. Most of these samples are systematic random samples, drawn by first choosing a random number (between 1 and k) and, starting from it, selecting every kth household in a numbered list of all households in the country. Samples include information from all individuals in the selected households. This sampling method has several features that we need to take account of when estimating population characteristics and performing statistical inference. An important feature of this sampling method is that primary sampling units are households and not individuals. Since we are mostly interested in the estimation of statistics for individual-level variables, these samples should be regarded as *cluster samples*. The probabilities of two members of a household of being included in the sample are not independent of each other and, as it will be discussed later, this might reduce the precision of our estimates. One sample

[1] Minnesota Population Center. Integrated Public Use Microdata Series, International: Version 7.1 [dataset]. Minneapolis, MN: IPUMS, 2018. https://doi.org/10.18128/D020.V7.1. These data comes from censuses and surveys collected by National Statistics Offices in each country. Brazil: Institute of Geography and Statistics; Costa Rica: National Institute of Statistics and Censuses; Dominican Republic: National Statistics Office; Ecuador: National Institute of Statistics and Censuses; Mexico: National Institute of Statistics, Geography, and Informatics; Panama: Census and Statistics Directorate; Trinidad and Tobago: Central Statistical Office; Uruguay: National Institute of Statistics.

(Mexico 2010) is drawn using geographic clustering: a fraction of the enumeration areas is randomly chosen and all households in those areas are sampled.

Another relevant feature of the sampling method is that it generates *implicitly stratified samples*. As explained by Cleveland et al. (2011), since the numbered lists of all households in these countries are often sorted according to small geographic areas, systematic sampling delivers a more even geographical distribution of households than is expected from simple random sampling. The samples from Brazil (2010) is the only one drawn using explicit stratification. In the case of Brazil 2010, population was divided in municipalities and a fraction of the households in each of them was randomly selected. Implicit or explicit stratification, as opposed to clustering, might reduce sampling variability and improve the precision of our estimates.

Finally, the sampling method also determines sampling weights for each individual in the sample. These weights are computed as the inverse of the probability of being selected as part of the sample and can be interpreted as the number of people in the population that each sampled individual represents. Sampling weights are critical for the estimation of population statistics from a sample, affecting estimators' bias and sampling variability.

A.1 Prevalence of Disability: Estimates.

Most of the statistics we estimate, such as the prevalence of disability in a given subpopulation or the proportion of high school graduates among people with some disability, take the form of a ratio (R) of two population totals (X and Y). We can formally define these population statistics in the following way (exploiting that most of the samples used in our analysis are organized in households and strata):

$$R = \frac{Y}{X} = \frac{\sum_{s=1}^{S} \sum_{h=1}^{H_s} \sum_{i}^{I_{hs}} y_{shi}}{\sum_{s=1}^{S} \sum_{h=1}^{H_s} \sum_{i}^{I_{hs}} x_{shi}} \tag{1}$$

where y_{shi} and x_{shi} are the values of variables Y and X for individual i in household h and stratum s; S is the total number of strata in the population; H_s is the number of households in stratum s; and i_{hs} is the number of individuals in household h in stratum s. For example, in the case of the prevalence of disability among a specific subpopulation, we measure the ratio between the number of people in that

subpopulation who report some type of disability (Y) and the total number of people in that subpopulation (X). In this case, variable y_{shi} would be a dummy indicator taking value 1 if the person belongs to the subpopulation of interest and reports a disability (and 0 otherwise); and variable x_{shi} would be a dummy indicator taking value 1 if the person belongs to the subpopulation of interest.

We estimate these proportions using data from a sample of households obtained from each stratum s. The number of households sampled in stratum s is defined as L_s. To perform this estimation, it is important to take account of the sampling weights implied by the sampling method. In most samples used for our estimations, households are selected in a way that each person represents the same number of people in the population and, therefore, sampling weights are flat (that is, equal for all people in the sample). In a few cases (Brazil 2010 and Mexico 2010), sampling methods are such that households in the population have different probabilities of being selected, and, therefore, different groups might end up being over- or under-represented. This occurs, for example, if a sample consists of a fixed number of households for each city in the country. In that case, people in smaller cities are more likely to be selected and will be overrepresented.

Our estimator for a population ratio ($\hat{R}$) is the ratio of the estimators for population totals ($\hat{Y}$ and $\hat{X}$). We estimate each population total with the Horvitz and Thompson (1952) estimator, using the sampling weights provided by IPUMS-International for each person in the sample:

$$\hat{Y} = \sum_{s=1}^{S} \sum_{h=1}^{L_s} \sum_{i}^{I_{hs}} \omega_{shi} \, y_{shi} \tag{2}$$

where w_{shi} is the sampling weight for individual i in household h and stratum s, and all other variables are defined as above. In those sampling designs in which there are unequal sampling probabilities, weighting each observation by its sampling weight eliminates the potential bias introduced by the over- and under-representation of different groups. To slightly simplify notation, we define the weighted total for variable y for household h in stratum s as:

$$y_{sh} = \sum_{i=1}^{I_{hs}} w_{shi} y_{shi} \tag{3}$$

The estimator for the ratio $\hat{R}$ is therefore:

$$\hat{R} = \frac{\hat{Y}}{\hat{X}} = \frac{\sum_{s=1}^{S} \sum_{h=1}^{L_s} y_{sh}}{\sum_{s=1}^{S} \sum_{h=1}^{L_s} x_{sh}} \tag{4}$$

In cases in which sampling weights are flat, the ratio estimator reduces to the ratio of the sample totals:

$$\hat{R} = \frac{\sum_{s=1}^{S} \sum_{h=1}^{L_s} \sum_{i}^{I_{hs}} y_{shi}}{\sum_{s=1}^{S} \sum_{h=1}^{L_s} \sum_{i}^{I_{hs}} x_{shi}} \tag{5}$$

While the Horvitz-Thompson estimator for the population total is unbiased, the ratio estimator ($\hat{R}$) is consistent but biased, since $E[Y/X] \neq E[\hat{Y}]/E[\hat{X}]$. However, the bias is small if sample size is large, the fraction of households sampled is large, and the population average of variable X is large and its standard deviation is small. The bias converges to zero as sample size increases to infinity. (Lohr, 1999)

Non-response and missing observations. We compute $\hat{R}$ using only respondents who provide information on both variables (that is, persons for whom y_{shi} and x_{shi} are not missing). We do not impute any value and we do not adjust sampling weights to account for non-response. The denominator ($\hat{X}$) might therefore vary between estimates that refer to the same subpopulation. Missing information could add an additional source of bias if the actual ratio (R) is different between respondents and non-respondents. In most surveys and for most questions, the number of missing observations is small, which limits the potential size of this bias.

A.2 Assessing Estimates' Precision: Standard Errors.

Estimates of population statistics obtained from random samples of the population are generally subject to sampling variability. For example, the estimate of the prevalence of disability in the population is likely to depend on the group of people randomly selected to be part of the sample and to vary if a different sample of the population is chosen. The magnitude of this variability depends on the sample size, on the sampling method, and on the underlying variability in the population (in an extreme-case scenario in which everyone in the population is equal, all samples deliver the same point estimate).

A usual measure of an estimates' sampling variability is the standard deviation of the estimates obtained from different samples, known as standard error. While computing the standard error requires having more than one sample, we can estimate its value using information from one sample. To do so in a precise manner, it is convenient to take account of different elements of the sampling design: sampling weights, clustering and stratification.

Sampling weights. Incorporating sampling weights into the estimation of population statistics generally changes not only the point estimates but also their standard errors. As mentioned by Kish (1992), unless unequal selection probabilities are purposely chosen to reduce sampling variability (that is, subpopulations with greater variance are oversampled), weighted estimators usually have larger standard errors than unweighted ones. In the case of Brazil 2010, there is explicit over-sampling of households in small municipalities, which might help to reduce sampling variance if variation in these municipalities is relatively large.

Clusters. The use of cluster sampling, which consists in dividing the population into disjoint groups (known as clusters) and randomly selecting some of those groups, might also impact sampling variability of population estimators. In all IPUMS-International samples we examine, there is household clustering: the population is divided in households, and a set of households is then randomly chosen. Samples include all individuals in the selected households. For equal sample sizes, sampling at the household level often leads to greater standard errors than randomly choosing individuals directly from the population (simple random sampling). This occurs when variation within households is smaller than variation between households, which is usually the case since members of the same household often share more traits and conditions among themselves than with other members of the population. Therefore, one random individual is likely to provide more information about the population (and lead to greater precision gains) than an additional member of a household in the sample. In an extreme-case scenario in which people are identical within households and different between households, adding additional members of a household carries no information and the standard error depends on the number of clusters and not on the sample size. This is the case for variables that are measured at the household level, such as poverty and access to infrastructure services.[2]

[2]In the case of Mexico 2010 there is also geographic clustering: a fraction of the enumeration areas is randomly chosen and all households in those areas are sampled. While we take account of household clustering, we do not incorporate geographic clustering in the estimation of standard errors. In the case that households are relatively similar within enumeration areas, this might lead

Stratification. Another feature of the sample design that affects sampling variability is stratification, which consists in dividing the population into disjoint groups (known as strata) and then drawing independent samples from each of these groups. Stratification may reduce sampling variability when population is relatively homogenous within strata and variation within strata is smaller than variation between strata. For instance, if there are two cities in the country, one with 1,000 inhabitants and other with 9,000 inhabitants, and 100 persons in each city are randomly selected, each of the cities is a stratum. If everyone in the smaller city has and reports some type of disability and none in the larger one does, stratifying by city reduces (and eliminates) sampling variability relative to randomly sampling 200 persons from the whole population.

Most IPUMS-International samples used for our analysis are implicitly stratified. As explained before, these samples are systematic random samples, drawn by first choosing a random number and, starting from it, selecting every kth household in a numbered list of all households in the country. Implicit stratification follows from the fact that lists with all households are often sorted according to small geographic areas and, therefore, systematic sampling delivers a more even geographical distribution of households than is expected from simple random sampling (Cleveland et al., 2011). Since many household characteristics are relatively homogenous within small geographic locations, this implicit stratification helps to reduce sampling variability. IPUMS-International provides a variable that, exploiting this logic and the geographical information available, defines pseudo-strata and helps to account for the implicit stratification imbedded in the sample design when computing standard errors (Davern et al., 2009; Cleveland et al., 2011).

To take account of sampling weights, clustering, and the (implicit) stratification of the sample, we estimate the standard error of our estimators assuming a stratified one-stage cluster sampling with replacement and unequal sampling probabilities (if sampling weights are not flat). In the case of the estimator for a population total $\hat{Y}$ (for example, the number of people with auditive disability in a given country), the estimator for its variance takes the form:

$$\hat{V}(\hat{Y}) = \sum_{s=1}^{S} \hat{V}(\hat{Y}_s) \tag{6}$$

to an overestimation of the precision.

where $\hat{Y}_s = \sum_{h=1}^{L_s} y_{sh}$ is the estimator for the total in stratum s and $\hat{V}(\hat{Y}_s)$ is the estimator for its variance:

$$\hat{V}(\hat{Y}_s) = L_s \sum_{h=1}^{L_s} \frac{(y_{hs} - \bar{y}_s)^2}{L_s - 1} \tag{7}$$

where $\bar{y}_s$ is the mean of the household totals for stratum s:

$$\bar{y}_s = \frac{1}{L_s} \sum_{h=1}^{L_s} y_{hs} \tag{8}$$

The standard error of the estimator is the square root of the estimated variance. The expression for $\hat{V}(\hat{Y})$ shows that the estimated variance of the estimator for a population total is the sum of the estimated variance within the different strata. This follows from the fact that sampling is independent across strata. As explained above, standard errors are small in cases in which the variation in the population is mostly explained by differences between strata. The estimator for the within-strata variance $\hat{V}(\hat{Y}_s)$ is derived assuming that sampling is performed with replacement and taking account of household clustering. In each stratum s, we assume we make L_s independent draws from the distribution of households (each household being drawn with the probability implied by its sampling weight divided by L_s) and estimate the variance of their distribution with the sample variance:

$$S(y_{sh}) = \sum_{h=1}^{L_s} \frac{(y_{sh} - \bar{y}_s)^2}{L_s - 1} \tag{9}$$

In our analysis, all statistics of interest are ratios of population totals (and, more precisely, proportions). To obtain the variance of the estimator for these ratios we use the so-called *Taylor linearization method.* The method consists in using the linear terms of the Taylor series expansion (around the true population value) to express the ratio as a linear function of population totals and then construct the variance of this linearized expression. The linearized expression for the estimator for ratio $\hat{R}$ is:

$$\hat{R} = R + \frac{1}{X}\hat{Y} - \frac{1}{X}R\hat{X} \tag{10}$$

The estimator for the variance of the ratio is therefore:

$$\hat{V}(\hat{R}) = \frac{1}{\hat{X}^2} \left\{ \hat{V}(\hat{Y}) + \hat{R}^2 \hat{V}(\hat{X}) - 2\hat{R}\hat{Cov}(\hat{Y}, \hat{X}) \right\} \tag{11}$$

where the estimated variance of the estimator for the population totals $\hat{Y}$ and $\hat{X}$ have the expression defined above, and the estimated covariance is:

$$c\hat{o}v(\hat{Y},\hat{X}) = \sum_{s=1}^{S} L_s \sum_{h=1}^{L_s} \frac{(y_{hs} - \bar{y}_s)(x_{hs} - \bar{x}_s)}{L_s - 1} \quad (12)$$

The estimation of the point estimates and standard errors is done using Stata, version 15.1. We follow IPUMS guidelines and use Stata's survey estimation commands (svyset and svy), defining variable "perwt" as sampling weights, variable "serial" as cluster identifier, and variable "strata" to define (pseudo) strata.[3]

A.3 Confidence Intervals.

We use the estimated standard errors not only to obtain a measure of the precision of our estimates but also to make statistical inferences on the population by constructing confidence intervals and testing hypothesis. Confidence intervals provide a range of values for a given population statistic (for example, prevalence of disability among children under 5 years old) that, based on the information from the available sample and its sampling method, might include the population value. The interval has an associated confidence level (expressed in percentage terms) that indicates the percentage of random samples obtained from the population for which the interval is expected to include the population value. The standard method for calculating the confidence interval is the so-called Wald method, which relies on approximating the distribution of the estimator with a Normal distribution centered at the population value. The bounds of the Wald confidence interval with confidence level 1-α are given by:

$$\hat{R} \pm z_{1-\alpha/2}\sqrt{\hat{V}(\hat{Y})} \quad (13)$$

where $z_{1-\alpha/2}$ is the $1 - \alpha/2$ quantile of the standard Normal distribution. The above formula is usually adjusted by replacing $z_{1-\alpha/2}$ by the corresponding quantile of the t-Student distribution with degrees of freedom determined by sample size and sampling method. While this is the standard and most widely used method, different studies have shown that its performance is poor for proportions: coverage levels

[3]IPUMS guidelines are available at: https://international.ipums.org/international/variance_estimation.shtml (accessed October 20th, 2020)

obtained from simulation exercises (that is, the percentage of confidence intervals that include the true value when obtaining different samples from some population) are smaller than nominal confidence levels, especially if true values are close to 0 or 1 or sample size is small (Dean and Pagano, 2015; Brown et al., 2001).

Since most of the population statistics we estimate are proportions, obtained by estimating the fraction of individuals with a certain attribute in a subpopulation, we employ an alternative method to compute confidence intervals: the Clopper and Pearson (1934) method, with the adjustments proposed by Korn and Graubard (1998) and Dean and Pagano (2015). The Clopper-Pearson method uses the binomial distribution to find the upper and lower bounds for the confidence interval. The lower (upper) bound is the lowest (highest) value of the probability of success -usually denoted by the letter p- such that the cumulative probability of the observed number of positive cases is $(\alpha/2)$ $1-\alpha/2$. Using the relationships between the binomial and the F distribution, the confidence interval bounds for the case of simple random sampling with replacement can be expressed Korn and Graubard (1998)

$$\mathrm{CI_{lb}} = \frac{\nu_1 F_{\nu_1,\nu_2,\alpha/2}}{\nu_2 + \nu_1 F_{\nu_1,\nu_2,\alpha/2}} \qquad \mathrm{CI_{ub}} = \frac{\nu_3 F_{\nu_3,\nu_4,1-\alpha/2}}{\nu_4 + \nu_3 F_{\nu_3,\nu_4,1-\alpha/2}} \tag{14}$$

where $\nu_1 = 2x$, $\nu_2 = 2(n - nx + 1)$, $\nu_3 = 2(x + 1)$ and $\nu_4 = 2(n - x)$; n is the sample size; x is the observed number of positive cases; and $F_{a,b,p}$ is the pth quantile of an F distribution with a and b degrees of freedom. We modify the Clopper-Pearson interval to make it applicable to a proportion estimated from complex survey data, as proposed by Korn and Graubard (1998) and Dean and Pagano (2015). The main modification is to replace the sample size (n) by the degrees-of-freedom adjusted effective sample size Dean and Pagano (2015):

$$n^{\star}_{df} = \frac{\hat{R}(1-\hat{R})}{\hat{V}(\hat{R})} \left(\frac{z_{1-\alpha/2}}{t_{d,1-\alpha/2}} \right)^2 \tag{15}$$

where $t_{d,1-\alpha/2}$ is the $1-\alpha/2$ quantile of the t-student distribution with d degrees of freedom. The survey design degrees of freedom (d) are calculated as the number of households in the sample minus the number of strata. The observed number of positive cases (x) is replaced by $\hat{R} \cdot n^{\star}_{df}$. These modifications seek to account for the additional variability produced by the sampling method (relative to simple random sampling) in the ratio estimator and in the estimated variance. Dean and Pagano

(2015) and Franco et al. (2018) evaluate the coverage and width of confidence intervals computed using alternative methods (including the standard Wald method and the adjusted Clopper-Pearson) via simulation. Their simulation exercises show that the adjusted Clopper-Pearson performs relatively well and provide better coverage than the standard Wald method, at the cost of sometimes being overly conservative.

A.4 Reporting Estimates: Suppression

For some statistics, sampling variability is large and point estimates might not be extremely informative. Furthermore, their estimated variance might also be imprecisely estimated, making confidence intervals unreliable. To avoid reporting such imprecise estimates, we follow the presentation guidelines proposed by the National Center for Health Statistics (NCHS) Data Suppression Workgroup (Parker et al., 2017). All the estimated rates satisfy the publication standard defined in those guidelines. The suppression rules are defined as follows:

Rule 1: If the sample size or effective sample size is less than 30, the estimate is suppressed.

The first rule indicates to suppress those estimates for which the relevant sample size (n) or the effective sample size ($n^\star$) are too small. The effective sample size is a measure computed when using data from a sample drawn with a sampling method other than simple random sampling and it is equal to:

$$n^\star = \frac{\hat{R}(1-\hat{R})}{\hat{V}(\hat{R})} \tag{16}$$

It can be interpreted as the sample size needed to obtain the observed level of precision (that is, the estimated variance) if the sample is drawn using a simple random sampling. To observe this, it is useful to note that, in the case of simple random sampling, the variance of the estimator $\hat{V}(\hat{R})$ can be estimated by $\hat{R}(1-\hat{R})/n$ and the effective sample size is equal to n. In the case that the proportion refers to some subpopulation, the sample size n is taken to be equal to the sample size for that subpopulation. If $\hat{R}$ is equal to 0 or 1, the effective sample size is undefined, and only the sample size should be used to verify compliance with this rule.

Rule 2: If the absolute confidence interval width is larger than or equal to 0.30, the estimate is suppressed.

The second rule tells to suppress those estimates for which the 95 percent confidence interval (estimated as described in the previous section) are wider than an arbitrary threshold of 0.30. The absolute confidence interval width is equal to the difference between the upper and the lower bound of the interval.

Rule 3: If the absolute confidence interval width is greater than 0.05 but lower than 0.30 and the relative confidence interval width is above 130%, the estimate is suppressed.

The third rule indicates to suppress those estimates for which the confidence interval width is moderate in absolute terms (between 0.05 and 0.30) but large relative to the point estimate. The relative confidence interval (used to implement the rule) is obtained by dividing the absolute width by the point estimate.

Rule 4: If design degrees of freedom are fewer than 8, the estimate is suppressed (unless a careful statistical review indicates the opposite).

The fourth rule tells to review those estimates for which the amount of design degrees of freedom (computed as the number of households minus the number of strata) is small. The number of degrees of freedom influences the precision of the estimated variance and of the confidence interval: with few degrees of freedom, the estimated variance is not precise and might lead to an unreliable confidence interval.

Rule 5: If $\hat{R} = 0$ or $\hat{R} = 1$, the estimate is suppressed (unless a careful statistical review indicates the opposite).

Finally, the last rule indicates to carefully review those estimates that are equal to 0 or 1. In those cases, confidence intervals are estimated using the sample size (n) and not the effective sample size ($n^{\star}$), which cannot be calculated. This might lead to underestimate the confidence interval width and overstate the estimate's precision.

A.5 Estimating Total Population with Disability: Extrapolation

In the Discussion, we report estimates by country for the total number of persons with a disability based on the estimated prevalence rates in two benchmark samples (Uruguay 2011 and Dominican Republic 2010). We construct the estimator in the following way: First, we estimate the prevalence of disability for different demographic groups in the benchmark sample using estimator (4). Second, we estimate the size of those demographic groups in each of the countries using estimator (2). Then, for each country, we estimate the number of people with a disability in each demographic group as the product of the prevalence rate in Uruguay 2011 (or Dominican Republic 2010) and the estimated size of the group's population. Finally, we sum across demographic groups and obtain the estimate for the total number of people with a disability in each country. Formally, the estimator is:

$$\hat{Y}_{\text{proj}} = \sum_{d \in D} \hat{X}_d \cdot \hat{R}_{d,\text{Uruguay 2011}} \tag{17}$$

where D is the set of demographic groups; $\hat{X}_d$ is the estimated population size for group d; and $\hat{R}_{d,\text{Uruguay 2011}}$ is the estimated prevalence of disability in group d using information from the Dominican Republic 2010 sample. As a robustness exercise, we also provide the estimates using the prevalence rates in Dominican Republic 2010:

$$\hat{Y}_{\text{proj}} = \sum_{d \in D} \hat{X}_d \cdot \hat{R}_{d,\text{Dominican Republic 2010}} \tag{18}$$

References

Brown, L. D., T. T. Cai, and A. DasGupta (2001). Interval estimation for a binomial proportion. *Statistical science*, 101–117.

Cleveland, L. L., M. Davern, and S. Ruggles (2011). Drawing statistical inferences from international census data. *IPUMS-International Working Paper* (20).

Clopper, C. J. and E. S. Pearson (1934). The use of confidence or fiducial limits illustrated in the case of the binomial. *Biometrika 26*(4), 404–413.

Davern, M., S. Ruggles, T. Swenson, J. T. Alexander, and J. M. Oakes (2009). Drawing statistical inferences from historical census data, 1850–1950. *Demography 46*(3), 589–603.

Dean, N. and M. Pagano (2015). Evaluating confidence interval methods for binomial proportions in clustered surveys. *Journal of Survey Statistics and Methodology 3*(4), 484–503.

Franco, C., R. J. Little, T. A. Louis, and E. V. Slud (2018). Comparative study of confidence intervals for proportions in complex sample surveys. *Journal of Survey Statistics and Methodology*.

Horvitz, D. G. and D. J. Thompson (1952). A generalization of sampling without replacement from a finite universe. *Journal of the American statistical Association 47*(260), 663–685.

Kish, L. (1992). Weighting for unequal pi. *Journal of Official Statistics 8*(2), 183.

Korn, E. L. and B. I. Graubard (1998). Confidence intervals for proportions with small expected number of positive counts estimated from survey data. *Survey Methodology 24*, 193–201.

Lohr, S. L. (1999). *Sampling: design and analysis. Brooks.* Cole Publishing Company. Pacific Grove, CA.

Minnesota Population Center (2018). Integrated public use microdata series, international: Version 7.1 [dataset]. `https://doi.org/10.18128/D020.V7.1`.

Parker, J., M. Talih, D. J. Malec, V. Beresovsky, M. D. Carroll, J. F. Gonzalez, B. E. Hamilton, D. D. Ingram, K. D. Kochanek, F. McCarty, et al. (2017). National center for health statistics data presentation standards for proportions.
