# Supplementary material for: Prevalence and correlates of disability in Latin America and the Caribbean: Evidence from 8 national censuses
Source: PLoS One. 2021 Oct 27;16(10):e0258825. doi: 10.1371/journal.pone.0258825 (PMC8550602; doi:10.1371/journal.pone.0258825)
Supplement: Attachment — Submitted filename: Response to Reviewers.docx [file pone.0258825.s019.docx]

**PONE-D-20-35261**

**Prevalence and Correlates of Disability in Latin America and the Caribbean: Evidence from 8 National Censuses**

**Reviewer #1:**

1. Definition of disability: Some of the censuses have used the Washington Group questions and recommendations. It will be advisable to use those questions and definitions when possible.

Thank you for the comment. We agree with the suggestion, and we use Washington Group (WG) questions when possible. In our responses to question 6 and 7 (below), we provide more details on how we operationalize the definition of disability.

2. Paragraph 3 of the introduction should be in the methodology or discussion.

Thank you for the suggestion. We have re-written the introduction and moved this information to the Discussion section in the paper.

3. Most of the information included in the introduction is part of the results or discussion. The authors will need to write a proper introduction, methodology, and results section. It is recommended that the authors re-write the introduction as an introduction. For now, it is the summary of different results, and it does not present the importance of this paper, the gap on the knowledge is not clear or the importance of this study for the LAC region. In addition, it is important to present what information exist on disability, what is the evidence on this topic for LAC and to have a clear discussion on what is the theoretical perspective that the paper uses.

Thank you for the suggestion. We have re-written the introduction, added emphasis on why this is an important question for the region, and made a stronger case for our contribution to the literature.

4. It is not clear why data from the Dominican Republic and Uruguay were used to compute specific country projections.

Thank you for pointing this out. We now explain in the text that both the Dominican Republic and Uruguay census instruments follow best practices in terms of high fidelity to implementing the Washington Group Short Set of questions. In contrast, other instruments diverged from recommendations by adding filter questions or terminology associated with the medical model. The projections results are very similar, independently of whether we use the Dominican Republic or Uruguay disability prevalence rates. Both countries have different demographic structures, so it is reassuring that the results of the projections are similar independently of the country used.

5. Although it is really important to present the definitions of disability included in each survey, it is recommended to reduce the length of that section and be more concrete about the definition used and what were the methodological decisions around this topic.

Thank you for the suggestion. We now move more quickly to our definition of disability. We then explain how the questions vary by country and how these variations might impact the disability prevalence estimates.

6. In addition, it is not clear if the harmonized data was used, why you were using the non-harmonized questions on disability? This is not clear in the text.

Thank you for the comment. We use IPUMS harmonized variables for employment and education, but not for disability. We have added a footnote clarifying this in the document. We reproduce the footnote below:

“Our disability indicator differs slightly from the harmonized variable provided by Minnesota Population Center (2018). We constructed our disability indicator to increase comparability across countries and adhere more closely to the WG recommendations on disability characterization. The differences between our variable and the harmonized variable are due to three reasons: First, in Brazil and Uruguay (but not in Trinidad and Tobago), the harmonized variable does not consider “some difficulty” as a disability. We consider “some difficulty” as a disability in all three countries. Second, some countries include a filter or a general question on disability status (e.g., Ecuador and Panama). The harmonized IPUMS variable takes the responses to this question to define disability. We construct our variable using responses to questions asking about difficulties or limitations in performing certain activities or tasks. Finally, we code respondents who responded positively to at least one of these questions as having a disability, even if they did not respond to all questions. The harmonized variable codes respondents who did not respond to a question as “unknown”.”

In Costa Rica and Trinidad Tobago, our disability indicator is equal to the harmonized variable proposed by IPUMS. In Mexico and the Dominican Republic, there are only differences in the characterization of a few missing observations. In Ecuador and Panama, we focus on the questions about functional limitations, while the IPUMS harmonized variable relies on answers to the filter or general question about disability status. Finally, in Brazil and Uruguay, there are differences regarding the threshold used to define disability and a few discrepancies in the characterization of missing values.

7. Also if you presented the recommendation made by the WG, why you did not follow them and instead follow recommendations that are not the ones with the largest support on the disability measurement? It is important to discuss what are the implications of how you measured/defined disability in the article.

Thank you for the comment. We construct a disability variable that maximizes comparability across countries, restricting to the WG questions when available. In the paper, we now explain our choice of the threshold (“some difficulty”) we use to define disability. The use of this threshold is aligned with the Washington Group’s recommendations and with methodological choices made by countries like Canada and the US.

8. It will be advisable to reduce the length of the computation of disability prevalence rates, this section can be shorter and more concrete providing only the information and formulas the reader needs. It is necessary that all analysis presented in the result section are introduced in the methodology, for example in the results the authors mention we estimate linear regression models for each country using a disability indicator as the dependent variable and a female indicator variable as a dependent variable. But this was never introduced in the methodology.

Thank you for the suggestion. We have simplified the Methods section by eliminating formulas and sending them to a technical appendix (in the supporting information). We have included the explanation of the regression analysis (previously in the results section) in the Methods section.

9. Statistical significant levels should be presented for all figures.

Thank you for the comment. All tables reporting regression results include levels of statistical significance. The figures (graphs) do not present confidence intervals for readability. However, we present 95-percent confidence intervals in the companion tables (available in the supporting information).

10. Why you selected to analyse the prevalence only for vision, hearing, motor, and cognitive impairments. This analysis also should be included in the methodology.

Thank you for the comment. We now include a reference to this analysis in the Methods section. Regarding the types of disabilities, we have clarified in a footnote that the WG short list of questions fails to capture psycho-social disabilities accurately. We focus on the prevalence of visual, hearing, motor, and cognitive disabilities, which are the types better covered in the countries in our sample.

11. Why only socioeconomic characteristics at the hh level were included? Why not to analyse level of education or employment of the person?

Thank you for the comment. Households are defined as being of low or high socio-economic status based on the education level of the head of household. However, statistics are otherwise computed at the individual level. When we examine employment rates by disability status and educational attendance or completion by disability status, the analyses are done using information at the individual level.

Reviewer #2:

1. Please limit the findings of this study to the results or discussion sections.

Thank you for suggestion. We have re-written the introduction section considering your suggestion and those of reviewer 1.

2. It will be very helpful if in this section to compare the disability status in LAC with other countries in the American Continent.

Thank you for the suggestion. We have added specific comparisons of our results with those in Canada and the US in the results section.

3. Please state in this section that estimates of the disability prevalence will be calculated for 2020 and 2050.

Thank you for your suggestion. We now refer to this in the section.

4. Please provide a rationale why Dominican Republican and Uruguay were used as reference countries.

Thank you for pointing this out. We now explain in the text that both the Dominican Republic and Uruguay census instruments follow best practices in terms of high fidelity to implementing the Washington Group Short Set of questions. In contrast, other instruments diverged from recommendations by adding filter questions or terminology associated with the medical model. The projections results are very similar, independently of whether we use the Dominican Republic or Uruguay disability prevalence rates for the simulation. Both countries have different demographic structures, so it is reassuring that the results of the projections are similar independently of the country used.

5. Please provide the missing information on disability for each country.

Thank you for the comment. We provide this information in “Table 1. Sample Size and Characteristics.” We have added a reference to the table in the “Data and methods” section.

6. How different or similar those participants not included in the analysis with those included.

Thank you for the comment. In most countries examined, the fraction of missing values is small (less than 1 percent of the sample). Only in Panama (1.76 percent), Trinidad and Tobago (9.91 percent), and Uruguay (6.07 percent), the fraction of missing values exceeds this threshold.

In the tables below, we compare some characteristics of individuals assigned a disability status (non-missing) with those considered “missing”.

In Dominican Republic (2010), “missing” and “non-missing” observations are relatively similar in terms of gender and age composition. Those respondents who have a “missing” disability status are more likely to be NIU or “not in the universe” of this question (that is, to be younger than ten years old) and to not report their employment status (and, therefore, less likely to report being employed, unemployed or inactive). Educational attainment is relatively similar across these two groups. Among those with “missing” disability status, there is a greater proportion of individuals NIU (that is, two years old or younger) and with less than primary completed.

In Trinidad and Tobago (2011), “missing” and “non-missing” observations are relatively similar in terms of gender and age composition. The differences are statistically significant, but the magnitudes are small. Those respondents who have “missing” disability status are more likely to be NIU (that is, to be younger than 15 years old) and to not report their employment status (and, therefore, less likely to report being employed, unemployed or inactive). There is also a higher fraction of individual NIU (14 years old or younger). Educational attainment is relatively similar across these two groups. Among those with “missing” disability status, there is a greater proportion of individuals NIU (although the inclusion criteria are not specified).

In Uruguay (2011), individuals younger than two years old are considered as “missing” in the disability questions (we have now clarified this in the text). This population accounts for 43.21 percent of the missing observations. Those respondents who have a “missing” disability status are more likely to be NIU (that is, to be younger than 12 years old) and to not report their employment status (and, therefore, less likely to report being employed, unemployed or inactive). In terms of educational attainment, there is a greater proportion of individuals NIU (3 years old or younger) among those with a “missing” disability status (and a smaller proportion in all other categories).

7. Please provide in the table or figure title for which year are the prevalence estimates (Table 2, Figure 3 to 6, Table 3 and 4, and Tables A.4 to A.14).

Thank you for the comment. We have added this information in the notes to all tables and figures.

8. This section it will benefit from a comparison with other continents. Where LAC stands in the prevalence of disability worldwide.

Thank you for the suggestion. We include a comparison with US and Canada and four African countries, all using the same threshold for defining disability and the Washington Group questions on functional difficulties.

9. A paragraph of strengths and study limitations is needed.

Thank you for the suggestion. We have added this paragraph to the conclusion.

10. On page 11, 2nd paragraph, line 7, the word of “incidence” should be replaced with “prevalence”.

Thank you for pointing this out. We have done it.
