# Supplementary material for: Prevalence and correlates of disability in Latin America and the Caribbean: Evidence from 8 national censuses
Source: PLoS One. 2021 Oct 27;16(10):e0258825. doi: 10.1371/journal.pone.0258825 (PMC8550602; doi:10.1371/journal.pone.0258825)
Supplement: Attachment — Submitted filename: Revision.docx [file pone.0258825.s020.docx]

**Prevalence and Correlates of Disability in Latin America and the Caribbean: Evidence from 8 National Censuses**

**Review**

The comments on the revised version of the paper are organized by sections below.

**Data and methods – Data sources and definitions**

In footnote 2 the authors state that the country selection is driven by data restrictions. It is useful to know which LAC countries that are included in IPUMS do not include disability information (other than Argentina).

I have several doubts about the missing data treatment. First, the missing proportions reported in table 1, refer to individuals with no data on disability, the ones that are not considered in the study? Or it reflects missing information in at least one disability variable? Second, the large proportion of missing data in Uruguay is outstanding. This seems to be caused by age-filters in the questions. As there is no information for children under 2, the authors should consider dropping the 0-2 population from the study (all the countries for comparability). Third, the information on differences between missing and non-missing information presented to reviewer 2 should be mentioned in the paper.

Table S2 show very important differences in the formulation of disability questions. This is the most important limitation of the paper. However, none of the countries follow the WG recommendation (<https://www.washingtongroup-disability.com/question-sets/wg-short-set-on-functioning-wg-ss/>). The compliance with the WG should be clearly stated by the authors. One issue that is not addressed in the paper is that the WG recommends that the answers to the limitation questions are graded, avoiding YES/NO formulations. The YES/NO formulation could discourage people with mild disability to declare disability, and lead to lower rates. Actually, this could explain (partially) the differences between countries observed in the paper. The two countries that ask for severity (Brazil and Uruguay) are the ones that show higher prevalence, followed by the YES/NO countries. The lowest prevalence occurs in countries that ask directly about disability (this is clearly stated by the authors).

In any case, I suggest that you directly refer in this section which are the countries that better adjust to WG recommendations (the ones used as reference for the projections).

Table S2: Wording of the Surveys' Disability-related Questions and Response Categories:

- In footnote 8 the authors note that Panama includes a filter for the general questions on disability. This is not clear in the S2 Table.
- In the third question for Trinidad and Tobago “Does the long-standing disability prevent (N) [the respondent] from doing any of the following? [Respondents indicate both the activity and its level of diffculty]” the authors do not list the activities.
- Notes on Table S2 should be revised.

**Prevalence of Disability: Estimates by country:**

The discussion of the prevalence country differences in terms of the formulation of the questions is not complete in my opinion, as it does not consider the severity options in the answers.

Line 284: the oldest group is 81+

Line 296: “fall close to” I think this may be stretching the interpretation (Brazil almost duplicates Canadian figures…). Actually, LAC figures are surprisingly high compared to the other 5 countries. Does this contradicts in any sense the socioeconomic gradient found within countries?

Type of disability: the age groups when analyzing the prevalence by type are somewhat strange. Why do the authors separate children in two groups? Why do they start the elderly group in 56?

The authors mention in the introduction the relevance of studying he association between poverty and disability. However, they study the socio-demographic gradient using the level of education of the household head. I understand that this comes from the lack of income and expenditure measures in the censuses data. This should be noted in the text. Also, the authors should consider trying a different specification for this socio-economic variable (ie. average educational years of the household adults) to explore the robustness of their results.

**Disability Gaps:**

I was at first stoke by the fact that Trinidad and Tobago had always the larger gaps, followed by Ecuador. These are the countries with lower prevalence (due to the formulation of the questions). The people that are identified as disabled are very different in these countries than in Brazil or Uruguay, they are probably the more severe. This should be at least clarified in the paper. In this sense, the note made in lines 427-432 about better and worse performing countries should be reconsidered.

**Projections.**

Regarding the selection of the countries on which to base the projections, the WG recommends that the answers to the limitation questions are graded, avoiding YES/NO formulations. I understand that the two countries that follow more closely the recommendation are Brazil and Uruguay. Dominican Republic´s questions allow for YES/NO answers. The authors should reconsider the inclusion of Dominican Republic rates for the projections or justify this based on different arguments. Also, they should justify he exclusion of Brazil.

Assumptions. One central assumption is not mentioned: that age- and gender-specific rates are assumed to be constant in time.
