# Supplementary material for: Prevalence and correlates of disability in Latin America and the Caribbean: Evidence from 8 national censuses
Source: PLoS One. 2021 Oct 27;16(10):e0258825. doi: 10.1371/journal.pone.0258825 (PMC8550602; doi:10.1371/journal.pone.0258825)
Supplement: Attachment — Submitted filename: Ressponse to reviewers.docx [file pone.0258825.s021.docx]

**Prevalence and Correlates of Disability in Latin America and the Caribbean: Evidence from 8 National Censuses**

**Reviewer #1:**

- Use the income classification of the country and not developing and developed countries.

*Thank you very much for the suggestion. We have modified the wording and now refer to measurement across high, middle, and low-income countries.*

- The introduction improved a lot, but it will benefit from including more information on previous research in the region.

*Thank you very much for the comment and the suggestion. We have added references to the following research for Latin America and the Caribbean.*

- *ECLAC. Regional Report on Measuring Disability. Overview of the disability measurement procedures in Latin America and the Caribbean. 2014.*
- *Pinilla-Roncancio, M. The reality of disability: Multidimensional poverty of people with disability and their families in Latin America. Disability and Health Journal. 2018.(11):398-404.*
- *United Nations. UN Flagship Report on Disability and Development. 2018.*
- The result section has improved a lot. However, sentences such as the one between lines 214 and 217 confuse the reader. Those sentences should be included in the discussion section.

*Thank you very much for the comment and the suggestion. We have moved the whole paragraph to the discussion section.*

- The WG questions should not be used for individuals younger than 15 years. The prevalences that the authors are presenting for individuals younger than 15 are affected by measurement error, and it is not recommended to estimate the prevalence of disability for this group using these questions.

*Thank you very much for this comment. Following this suggestion and that of another reviewer, we have dropped the 0- to 2-year-old population from our analysis and report prevalence rates for individuals aged three years and older. We have included a table in the supporting information that reports prevalence rates among individuals aged 15 years and older in response to your comment. We have also included a sentence referring to a specific module (WG-UNICEF) to measure functional difficulties among children more accurately. However, this module has not yet been widely implemented in censuses in the region, and, therefore, we rely on the information available to provide an estimate for this population.*

*As the paper shows and the literature has discussed, disability prevalence estimates -for all age groups- are strongly affected by the survey instrument used to measure disability. We are aware of this and discuss it throughout the paper.*

- The discussion needs still a lot of work; in its current state, it only summarizes what the authors did but not discusses the results.

*Thank you for your comment. The discussion compares the results of our estimate with countries outside the region (as you suggested) and, looking ahead, stresses the role of an aging LAC population on the prevalence of disability in the region. This dynamic is key in motivating the projections for LAC, which we see as the main point of the discussion section. The conclusion summarizes our results and provides policy recommendations.*

- Given the different definitions of disability and that some countries use the WG questions and a severity scale, it is recommended to estimate the prevalence using other categories.

*Thank you very much for this comment. For completeness and comparability, we now include the estimated overall prevalence rates considering only individuals who reported “a lot of difficulty” or more as having a disability. Using this alternative definition of disability, overall disability prevalence rates fall from 24.9 to 6.9 percent in Brazil (2010), from 4.5 to 2.4 in Trinidad and Tobago (2011), and from 17 to 5 percent in Uruguay (2011).*

- In school attendance, it is essential to acknowledge that school attendance would vary between impairments even if the measure of disability were correct.

*Thank you very much for this comment.* *In discussing the gaps in education, we have added the following footnote: “The CRPD does not contemplate that severity of difficulty should be correlated with inclusion. In practice, however, the estimated gaps may be reflecting differential access based on the gradation of disability. Differences in measurement can also affect results across countries*.”

*In response to this comment and that of another reviewer, we have also included an additional table (Table S11 in the supporting information) estimating the disability prevalence rates and education gaps for the four different types of disabilities we consider: vision, motor, hearing, and cognitive. We observe that disability gaps in school attendance vary between different types of disabilities. The average gap is larger for children with cognitive and motor disabilities, followed by those with hearing disabilities. In children with vision disabilities, we observe a positive but smaller gap among primary school-age children and no systematic gap among secondary school-age children. The patterns are similar (but more pronounced) when comparing school completion gaps. We have included this discussion in the paper.*

**Reviewer #3: Prevalence and Correlates of Disability in Latin America and the Caribbean: Evidence from 8 National Censuses**

Review
The comments on the revised version of the paper are organized by sections below.

**Data and methods – Data sources and definitions**

- In footnote 2 the authors state that the country selection is driven by data restrictions. It is useful to know which LAC countries that are included in IPUMS do not include disability information (other than Argentina).

*Thank you very much for your comment. We have clarified the inclusion criteria for the study and that the census data for Argentina 2010 and Bolivia 2012 are available in IPUMS but do not include data on disability.*

- I have several doubts about the missing data treatment. First, the missing proportions reported in table 1, refer to individuals with no data on disability, the ones that are not considered in the study? Or it reflects missing information in at least one disability variable? Second, the large proportion of missing data in Uruguay is outstanding. This seems to be caused by age-filters in the questions. As there is no information for children under 2, the authors should consider dropping the 0-2 population from the study (all the countries for comparability). Third, the information on differences between missing and non-missing information presented to reviewer 2 should be mentioned in the paper.

*Thank you very much for your comment. The missing proportions reported in Table 1 refer to individuals who are considered as missing for our analysis. We have now clarified this in the document (footnote 3). We have also extended the description of the missing data treatment to make it more transparent for the reader.*

*Following yours and another reviewer’s comment, we have decided to drop the 0-2 population from the analysis. We have re-estimated all prevalence rates and now present figures considering only individuals aged 3 and older. This change has reduced the proportion of missing values, especially in Uruguay.*

*We re-did the analysis presented to Reviewer 2 (first revision), considering only individuals aged three and older. Following your suggestions, we now present the tables comparing characteristics of individuals who were assigned a disability status (non-missing) with those considered “missing” for the Dominican Republic, Trinidad and Tobago, and Uruguay (where missing values exceed 1 percent of the observations) in Table S9 (in the supporting information).*

*We do not observe major differences in age and sex composition between “missing” and “non-missing” observations in any country. The differences are statistically significant, but the magnitudes are relatively small. Those respondents who have “missing” disability status are more likely not to report their employment status (and, therefore, less likely to report being employed, unemployed, or inactive) and their education status.*

- Table S2 show very important differences in the formulation of disability questions. This is the most important limitation of the paper. However, none of the countries follow the WG recommendation (<https://www.washingtongroup-disability.com/question-sets/wg-short-set-on-functioning-wg-ss/>). The compliance with the WG should be clearly stated by the authors. One issue that is not addressed in the paper is that the WG recommends that the answers to the limitation questions are graded, avoiding YES/NO formulations. The YES/NO formulation could discourage people with mild disability to declare disability, and lead to lower rates. Actually, this could explain (partially) the differences between countries observed in the paper. The two countries that ask for severity (Brazil and Uruguay) are the ones that show higher prevalence, followed by the YES/NO countries. The lowest prevalence occurs in countries that ask directly about disability (this is clearly stated by the authors). In any case, I suggest that you directly refer in this section which are the countries that better adjust to WG recommendations (the ones used as reference for the projections).

*Thank you very much for the comment. We have clarified that no census strictly adhered to the WG recommendations and have described the potential measurement issues in the section, including how binary (Yes/No) formulations can miss less severe disabilities.*

- Table S2: Wording of the Surveys’ Disability-related Questions and Response Categories:
- In footnote 8 the authors note that Panama includes a filter for the general questions on disability. This is not clear in the S2 Table.

*Thank you very much for the comment. The Panama 2010 survey includes a general question on disability, which is included last in the Table S2:* “What type of physical or mental disability do you have?” *We have clarified the expression in footnote 8 to avoid confusion:* “Second, some countries include a filter question (e.g., Ecuador) or a general question (e.g., Panama) on disability status.”

- In the third question for Trinidad and Tobago “Does the long-standing disability prevent (N) [the respondent] from doing any of the following? [Respondents indicate both the activity and its level of diffculty]” the authors do not list the activities.

*Thank you very much for pointing this out. We have revised it.*

- Notes on Table S2 should be revised.

*Thank you very much for pointing this out. We have revised the notes.*

**Prevalence of Disability: Estimates by country:**

- The discussion of the prevalence country differences in terms of the formulation of the questions is not complete in my opinion, as it does not consider the severity options in the answers.

*Thank you very much for your comment. We have included this in the discussion and added a reference to the research that finds that when a binary scale is used, rather than one capturing gradations of difficulties, less severe cases of disability are not reported (Altman et al., 2014).*

- Line 284: the oldest group is 81+

*Thank you for your comment. The oldest group in Figure 2 is 81+. In Figure 5 and Table S5 the oldest group is 56+. We were mistakenly indicating that the oldest group was 55+.*

- Line 296: “fall close to” I think this may be stretching the interpretation (Brazil almost duplicates Canadian figures…). Actually, LAC figures are surprisingly high compared to the other 5 countries. Does this contradicts in any sense the socioeconomic gradient found within countries?

*Thank you for your comment. The age-specific rates of disability for 65–74-year-olds are higher in LAC than in Canada or the US, using a similar application of the WG questions. This pattern aligns with the socioeconomic gradient found within countries.*

*Then, the paragraph (now in the discussion section) reports the overall prevalence of disability for four African countries (Mitra, 2018). These rates average 13.5 percent, slightly higher than the Dominican Republic 2010 (12,4 percent in the whole population and 13 percent among those aged three and older) and lower than Uruguay 2011 (17 percent among those aged three and older) -the two countries we use as a benchmark for applying a set of questions close to the WG recommendations-. When considering prevalence among the entire population, the age structure may offset socioeconomic influences. As we mention in the paper, the difference in prevalence between countries may be due to, among other things, differences in the demographic structure of the countries, and these four African countries have a relatively young population.*

**Type of disability:**

- The age groups when analyzing the prevalence by type are somewhat strange. Why do the authors separate children in two groups? Why do they start the elderly group in 56?

*Thank you very much for your comment. We use age groups for children that correspond to standard age groups for different levels of schooling as used by UNICEF. Although standards vary across countries, ages 3-5 is the standard age for preschool, 6-11 for primary school, and 12-17 for secondary school. Retirement ages are younger in LAC, so we analyze the older population starting with 56 and older. We acknowledge that there is no strict definition of the elderly population. Some publications use 60 or 65 as the cut-off age.*

- The authors mention in the introduction the relevance of studying he association between poverty and disability. However, they study the socio-demographic gradient using the level of education of the household head. I understand that this comes from the lack of income and expenditure measures in the censuses data. This should be noted in the text. Also, the authors should consider trying a different specification for this socioeconomic variable (ie. average educational years of the household adults) to explore the robustness of their results.

*Thank you very much for the comment. As you correctly indicate, we use the level of education of the household to study the socioeconomic gradient due to the absence of comparable and reliable measures of household income, assets, or expenditures. We now indicate this in the text (data section). In addition, in the introduction, we have modified the sentence* “We then examine the association between disability and poverty” *to* “We then examine the socioeconomic gradient of disability,” *which describes what we do more accurately.*

*We have explored the alternative specification to our metric you suggested. As reported in the table below, household head education is highly correlated with the average education of household adults. This result is intuitive: educational assortative mating probably leads to similar educational levels within couples. Given the high correlation between these metrics, we prefer to keep our chosen metric as it is intuitive and -as opposed to the average- provides clear and well-defined categories.*

| Sample | Corr: Head Education vs. Avg. Ed. Household Adults |
| --- | --- |
| Brazil 2010 | 0.8522 |
| Costa Rica 2011 | 0.8396 |
| Dominican Republic 2010 | 0.8336 |
| Ecuador 2010 | 0.7550 |
| Mexico 2010 | 0.8313 |
| Panama 2010 | 0.8603 |
| Trinidad & Tobago 2011 | 0.7832 |
| Uruguay 2011 | 0.9408 |

**Disability Gaps:**

- I was at first stoke by the fact that Trinidad and Tobago had always the larger gaps, followed by Ecuador. These are the countries with lower prevalence (due to the formulation of the questions). The people that are identified as disabled are very different in these countries than in Brazil or Uruguay, they are probably the more severe. This should be at least clarified in the paper. In this sense, the note made in lines 427-432 about better and worse performing countries should be reconsidered.

*Thank you very much for your comment. We have added the following footnote in response to it: “The CRPD does not contemplate that severity of difficulty should be correlated with inclusion. In practice, however, the estimated gaps may be reflecting differential access based on the gradation of disability. Differences in measurement can also affect results across countries.”*

**Projections.**

- Regarding the selection of the countries on which to base the projections, the WG recommends that the answers to the limitation questions are graded, avoiding YES/NO formulations. I understand that the two countries that follow more closely the recommendation are Brazil and Uruguay. Dominican Republic´s questions allow for YES/NO answers. The authors should reconsider the inclusion of Dominican Republic rates for the projections or justify this based on different arguments. Also, they should justify he exclusion of Brazil.

*Thank you for your comment. We have taken your suggestion on board regarding the projections: Our baseline estimates come from Uruguay only. We also report estimates using sex- and age-specific rates from the Dominican Republic as a robustness exercise (in the supporting information). The Dominican Republic also tracked the WG recommendations closely (despite the use of Yes/No responses), and it is at a different stage in the demographic transition relative to Uruguay. We leave Brazil out because of the unusually high prevalence of vision disabilities. We have now clarified this in the document.*

- Assumptions. One central assumption is not mentioned: that age- and gender-specific rates are assumed to be constant in time.

*Thank you very much for pointing this out. You are entirely right. We have revised the text accordingly.*

**Reviewer #4: Review report for: Prevalence and Correlates of Disability in Latin America and the Caribbean: Evidence from 8 National Censuses**

**Introduction**

- I think that the paper could benefit so much if the authors deepen in the motivation, the discussion of their main empirical findings, the policy implications, and the recommendations to better measure prevalence.

*Thank you very much for the comment. We strengthened the motivation in the introduction, as suggested. Based on comments from a first-round revision of the paper by other reviewers and the editor, we had already modified the style and format to align it with that of PLOS One. These previous comments required us to move the description of the results and their discussion to the respective sections of the paper, leaving only motivation and related literature for the introduction*

- It would be useful to present the main results of the paper in the introduction. Also, it would be necessary to relate them with the main contribution of the paper mentioned in the introduction and with the previous literature. I think that the author may use the introduction to explain how their finding contributes to the existing knowledge.

*Please see the previous response. We were asked to exclude the results from the introduction.*

- I recommend to the authors to present a discussion on what is the theoretical perspective that the research adopts to address this issue. In addition, it is relevant to present the previous evidence on this topic for LAC.

*Thank you very much for the comment. We have included additional references to previous research in LAC. Our conceptual framework follows the social model of disability (which incorporates the interaction between a person’s impairment and the barriers to functioning in their environment), which is explained in the second section of the paper. We refrain from discussing it in great detail in the introduction following the style recommendations of PLOS ONE. However, we now clarify: “*Seven of the countries measured disability using a version of the Washington Group questions that were formulated to measure disability consistent with the social model in the CRPD, although none strictly implemented the recommendations.”

- The paper said (lines 18-19), “We then examine the association between disability and poverty…”. However, this issue is not addressed in the next sections. I understand that the authors have not microdata about household income and poverty levels at the household (or individual) level. To explore that association maybe they could use secondary database (e.g. National Household surveys) and analyze that relationship at regional levels. More in general, the regional heterogeneity within countries is not addressed, and could be interesting to extend the analysis.

*Thank you very much for the comment. As you correctly mention, we use the level of education of the household to study the socioeconomic gradient due to the absence of comparable and reliable measures of household income, assets, or expenditures. We now make this explicit in the data section of the paper. In addition, in the introduction, we have modified the sentence* “We then examine the association between disability and poverty” *to* “We then examine the socioeconomic gradient of disability,” *which describes what we do more accurately.*

*While we agree with you that examining regional heterogeneity within countries is an interesting analysis, we believe it goes beyond the scope and goal of the paper.*

- I think that the motivation could improve if the authors justify the novelty of the selected countries. In the current version of the manuscript, the inclusion criteria are based on data availability. The set of countries seems to be heterogeneous, but there is not a systematic discussion about this (demographic, cultural, institutional differences). Maybe we could learn anything about that heterogeneity.

*Thank you for your comment. We now present information on income per capita and population structure of the different countries in Table S14 (included in the supporting information). The countries represent a wide range of levels of economic development and age structures. For example, in 2010, the Dominican Republic had a GDP per capita of 5600 dollars, and only 12 percent of the population was over the age of 55. Uruguay had about twice as large a GDP per capita and a share of the population over 55 as the Dominican Republic. We address this in the text of the paper, but -for stylistic purposes explained earlier- not in the introduction.*

- I think that the motivation could be related to the potential policy implication associated with the main results. Could you explain in more detail the policy implications of your findings? (Maybe this issue could be considered in the conclusion section)

*Thank you for this comment. We have included references to more specific policy implications in the conclusion section.*

- To advance in the second contribution, it could be useful to incorporate some specific recommendations about disability questions that could be considered in the future census of these countries.

*Thank you for your comment. We address this already in the conclusion section*.

**Data and method**

- In lines 65 and 66 the text said “For our analysis, we construct a disability variable that maximizes comparability across countries, restricting to the WG questions when available.” Then the Table S2 provides details about the questions to measure disability in each census. It could be useful to include in this Table the WG questions as reference. This could help to describe the synthesis about the comparability across countries.

*Thank you very much for this suggestion. We have included the Washington Group Short Set on Functioning (WG-SS) in Table S2.*

- When the authors introduce the socioeconomic variables, they could mention when they refer to the household head.

*Thank you very much for pointing this out. We have added a paragraph in the data section of the paper indicating that we use the household head’s educational level to approximate the household’s socioeconomic situation.*

**Estimation method**

- Is it possible to better link the parameters of equations 1 and 2 with the results about the average prevalence by groups?

*Thank you very much for the comment. In equation 1, the coefficient of interest (β) is (approximately) the population-weighted average of the sex gap in the disability prevalence rates across cohorts. We have added this clarification in the paper. The coefficient of interest in equation 2 refers to the sex gap in employment and educational attainment between individual with and without a disability. We do not find better ways to link it with average prevalence rates by groups.*

- Equation 2 includes cohort fixed effect. However, the note in Table 3 includes “age, fixed effect as dependent variables”. I think that is the same variable, but it could be more appropriate to use a unique label. I identified a similar situation in other parts of the text (e.g. line 174). A minor issue, in the note of Table 3 the authors refer to the fixed effect as a dependent variable. I think that is a typo mistake.

*Thank you very much for pointing this out. As you correctly indicated, cohort and age fixed were the same (independent) variables. We have made the corrections accordingly.*

- In this section, the authors could include the reference to Section 5 of the Appendix, entitled “Estimating Total Population with Disability: Extrapolation. A minor issue: In this section, equation 17 refers to the Dominican Republic, but it does not consider Uruguay.

*Thank you very much for this suggestion. We have included the reference to Section 5 of the Appendix, both in the data and discussion sections. We have also modified that section to include Uruguay.*

**Results**

- The first general comment is that this section could improve if the authors advance in the interpretation of the results, establishes some hypotheses, and explore the ways to strengthen dialogue with the previous papers.

*As with the introduction, in the first round of reviews, we were asked to relegate the discussion of results to the discussion sections for style purposes. In keeping with the style and format of PLOS ONE, we have not followed your recommendation.*

- Could you explain the origin of the disability gap by sex? I expect that for these countries the average male life expectancy is lower than females. Is the origin of the sex gap that men with disabilities tend to die younger than the other men or than women with disabilities? Is it that there are more women between adults older which introduces persons with new disabilities? Evidence about these issues could be policy implications.

*Thank you for this question. We agree that it has relevant policy implications. Women’s higher life expectancy rates contribute to a higher representation of women among people with disabilities, particularly among the elderly population. When we control for age in the regressions, we find that in over half the countries, prevalence rates for disability are higher for males under age 30, while women have higher prevalence rates at older ages.*

- Table 2/Figure 1: Brazil shows the highest prevalence. Could you suggest any hypothesis to explain that result?

*To the extent that Brazil applied questions similar to the recommended ones by the WG rather than a strictly medical approach, a higher prevalence would be expected. The prevalence rates for motor, hearing and cognitive disabilities, as shown in appendix Table S4.1 are similar to those for Uruguay (6.05, 5.09, and 1.36 vs. 6.64, 3.75, and 2.86, respectively). Where Brazil stands out is the high prevalence rate for visual disabilities, which is twice that in Uruguay (18.8 vs. 9.82). We can only speculate, but two plausible explanations come to mind. The first is a problem with access to assistive technology (glasses) in Brazil. The second is a potential problem with the enumerators’ training, such that they did not always inquire about difficulties even wearing glasses in Brazil.*

- Table 3: It could be useful to include in the Table the prevalence per group and country to identify the magnitude of the gap.

*Thank you very much for the suggestion. We have modified Table 3 to include the overall disability prevalence rate per group and country.*

- The household head analysis considers the education level. This analysis could be introducing a cohort bias because those people with primary incomplete could be older.

*Thank you very much for this comment. We share your concern, which is one reason we also present the analysis by age brackets (Figure 6). It is reassuring that a strong socioeconomic gradient is observed in all different age groups. [See the table in a comment below.]*

- Table 4. Please explain how this table considers the educational lag of individuals.

*Thank you for this comment. We have estimated net primary and secondary attendance rates, which measure attendance at the standard ages of primary and secondary school but not attendance at a specific level of education. As such, these measures do not necessarily reflect grade delays. The grade delays would be more likely to be reflected in the secondary completion rates where being three or more years behind peers is a high-risk factor for school drop-out.*

- Table 4 It would be useful to comment in this case whether there are differences by gender. Idem comment for Table 5.

*Thank you very much for this suggestion. We have included the estimates of education and employment disability gaps by sex in tables S10 and S11 (included in the supporting information). We have also added a short description of the results by sex in the paper’s results section.*

- In lines 286 and 287 the text states “In all age groups for practically all countries, the prevalence of disability is significantly higher in households with low education levels.” I not sure that this result is confirmed for persons under six years old.

*Thank you very much for your comment. Please note that we have now dropped individuals aged 0 to 2, and, therefore, estimates for persons under six years old have changed. We believe the statement is accurate. The table below presents the ratio of the disability prevalence between individuals in households whose head has not completed primary and individuals in households whose head has a university degree for different age brackets. Except for Trinidad and Tobago, disability prevalence rates for children aged 3 to 5 in households whose head has not completed primary is substantially higher than in the most educated households.*

***Disability Prevalence Ratio: Individuals in households with low vs. high educated households’ heads.***

|  | **3 and older** | **3 to 5** | **6 to 17** | **18 to 54** | **55 and older** |
| --- | --- | --- | --- | --- | --- |
| Brazil | 1.61 | 1.80 | 1.34 | 1.58 | 1.60 |
| Costa Rica | 2.19 | 1.91 | 1.66 | 1.66 | 1.93 |
| Dominican Rep. | 1.46 | 1.47 | 1.10 | 1.11 | 1.35 |
| Ecuador | 2.86 | 1.94 | 2.07 | 2.96 | 2.05 |
| Mexico | 3.64 | 1.47 | 1.63 | 3.18 | 2.43 |
| Panama | 1.92 | 1.30 | 1.34 | 1.69 | 1.67 |
| Trinidad & Tobago | 2.35 | 0.61 | 1.06 | 1.61 | 3.23 |
| Uruguay | 4.00 | 1.85 | 3.82 | 3.57 | 2.33 |
| **Average** | **2.50** | **1.54** | **1.75** | **2.17** | **2.07** |

*Note: Authors’ estimations. The figures report the ratio of the disability prevalence rates between individuals in households whose head has not completed primary and individuals in households whose head has a university degree for different age brackets.*

- In order to interpret the results in Table 4, it would be useful to consider more contextual information. For example, the authors could report whether there are differences in the education supply for children with disabilities.

*Thank you for your suggestion. There is a wide variation in the approach to the education of children with disabilities across the eight countries. Our intention in calculating the gaps in Table 4 is to encourage policymakers to examine in detail the institutional context where gaps are particularly large. Given that there are eight countries in our sample, collecting and reporting this information will take us well beyond the scope of this paper.*

- Table 5. Why you selected to analyze only the employment rates? Participation and unemployment rate might provide complementary information, and provide new insights for policy implications.

*We believe the most relevant and easiest-to-interpret margin is whether individuals with disabilities are employed or not and how their employment rate compares with that of people with no disabilities. Following the suggestion from other reviewers, we have expanded the analysis on employment gaps, distinguishing by sex and type of disability. While still relevant, we consider adding the study of participation and unemployment rates would result in an excessively long section.*

- Line 364 said: “The variability between countries in the employment gap, as in disability prevalence rates and education gaps, may reflect multiple factors (including definition and recording of disability).” This statement is so general that it questions the results previously presented. At this point, as a reader, emerges the question about what we have to learn from the study and what its original contribution is. Moreover, I think that this statement seems contradictory with the comments of the first paragraph in the conclusion section.

*Thank you for your comment. The entire paragraph reads:*

“The variability between countries in the employment gap, as in disability prevalence rates and education gaps, may reflect multiple factors (including definition and recording of disability). Still, looking at the region’s best performers’ approaches might provide relevant examples and guidance to countries lagging.”

*It suggests there are many reasons for differences in outcomes across countries, but countries that do better may be doing something right that is worth exploring. Identifying the reason behind these differences is beyond the scope of the paper: it would not be feasible to look at employment, education, health, social protection, and disability policies for eight countries. However, we do hope that our research motivates other researchers to investigate these differences more closely.*

**Discussion**

- Please provide a brief discussion about the validity of the assumptions presented in lines 387 to 391. Note that the paper does not present the differences between countries (for example, in terms of demographic structure).

*Thanks for your comment. Our projections rely on three assumptions. First, the questions formulated in Uruguay correctly captured the disability status of the population. Second, the age and gender-specific rates are constant over time. Third, for each age-sex group, differences in the observed prevalence of disability between countries result only from the variation in the survey questions’ approach and interpretation. We do not question the validity of the population projections by the UN.  The first assumption seems reasonable. The last two are more nuanced as countries in the region are in different stages of economic development. However, using Uruguay as a benchmark has the advantage that, as one of the most developed countries in the region and with the best social protection network, it is likely to provide a lower bound to the incidence of disability for the rest of the region. Of course, our estimates are* ceteris paribus *in the sense that they cannot account for any significant reform on inclusion policies that may affect the region. In the parlance of economists, our projections are subject to the Lucas’ critique.*

- Information from the previous census could be useful to assess the validity of the projections and assumptions included in the analysis (It is just an idea; I do not expect an answer on this point.).

*Thank you for your comment and the interesting suggestion.*

- How these projections of people living with a disability may affect the need for resources for inclusion policies? Given that projection, the author could provide any idea about the magnitude of the fiscal effort that would imply maintaining current inclusion policies.

*Thank you for your suggestion. Given that there are eight countries in our sample, collecting and reporting this information will take us well beyond the scope of this paper.*

**Reviewer #5:**

**In Table 2**

- The prevalence of disability varies widely between countries. How are these differences interpreted in the absence of a conceptual framework? Since an effort is made to reconcile the various indicators used to approach the question of disability, how much does the heterogeneity in the questions contribute to the differences between countries? Is it not possible to decompose these results? Or perhaps it is the demographic structures of the countries that explain these results? I am struck by the prevalence in the case of Brazil, given its age structure.

*Thank you for your comments. The conceptual framework of the paper is indeed the social model of disability as embodied in international frameworks. As we explain on page 3 of the paper:*

*“In recent decades, the notion of disability has evolved from a strictly medical concept to incorporate the interaction between a person’s impairment and the barriers to functioning in his or her environment. According to this approach embodied in the CRPD and the WHO’s International Classification of Functioning, Disability, and Health, a person’s disability is defined as a dynamic interaction between an impairment and external factors; on its own, a health condition or impairment is not a disability.”*

*In this framework, the differences in the incidence of disability across countries can be explained by different factors. The first factor is the incidence of impairments, which may be linked to the country’s demographic structure (particularly age). The second factor is the environmental barriers to functioning, which at the individual level, can be affected by income (which we proxy with the education of the head of household) and, at an aggregate level, by the access and accommodations that a country provides for people with impairments. A third factor, which we address extensively in the paper, is related to the measurement of disability. As we explain in the paper, the WG questions are explicitly designed to capture the social model of disability and avoid stigmatizing language that may suppress reporting. However, not all countries have yet fully incorporated these questions.*

*The paper establishes common patterns in age, gender, and socioeconomic status (education of the household head) that reflect the fundamental concepts of the social model. Nonetheless, measurement issues continue to affect the comparability across countries. We make a robust push for countries to implement common measurement tools, which will eventually allow us to focus on the critical issues of impairments and barriers rather than on the more “academic” issue of measurement.*

*Following your comment, we have made these contributions more salient in the paper. However, as the paper is written for the broader audience of PLOS ONE and its main goal is to establish stylized facts about disability in LAC, we do not include a conceptual framework section (as one would expect, for example, in an economics paper).*

- Is there an explanation for the absence of significant differences between men and women in Trinidad and Tobago and Mexico?

*Thank you for your comment. The objective of the paper is to establish stylized facts about disability prevalence in LAC. Explaining every difference between the eight countries is beyond the scope of the paper as it will require looking at employment, education, health, social protection, and disability policies for eight countries. We do hope, however, that our research motivates other researchers to investigate these differences more closely.*

**In Table 3**

- The prevalence of disability according to age groups shows different behaviors between countries. The only countries with similar behavior are Trinidad and Tobago with Costa Rica and Brazil with the Dominican Republic. How are these heterogeneous trajectories explained?

*Thank you for your comment. As indicated in response to the previous comment, the paper’s objective is to establish stylized facts about disability prevalence in LAC. Explaining the heterogeneous trajectories between the eight countries would be a highly speculative task and is beyond the scope of the paper. We do hope, however, that our research motivates other researchers to investigate these differences more closely.*

- Is it not possible that demographic structure is at play in within the age groups? Wouldn’t the life expectancy of men and women affect the results, in a heterogeneous way, for those over 65 years of age? To observe this, within age groups, you could generate interactions of sex with age.

*Thank you very much for this comment. This would undoubtedly be an issue if we did not include cohort (i.e., age) fixed effects in the regression model. The inclusion of cohort fixed effects (for each year) helps us account for the systematic difference in the demographic structure across sexes within age groups. The resulting coefficient of interest (β) is (approximately) the population-weighted average of the sex gap in the disability prevalence rates across cohorts. In other words, the exercise provides an estimate that results from comparing men and women of the same age and averaging across the different ages.*

**In Tables 2 and 3**

- Couldn’t the differences between countries be the consequence of the prevalence of different types of disability in each of the countries? Is it impossible to make the same estimates according to the types of disability (or excluding the types of disabilities one by one)?

*Thank you very much for this question. In Table S4 and Figure S1 (in the supporting information) we present the prevalence rates of different types of disability by country and sex for different age groups. The table and the graph below summarize the information (considering all individuals aged three and older). The pattern is consistent across countries: vision and motor disabilities are the most prevalent, and hearing and cognitive disabilities are the least prevalent in all countries.*

***Table: Disability Prevalence Rates by Type of Disability (Ages 3 and older)***

|  | **Motor** | **Vision** | **Hearing** | **Cognitive** | **Overall** |
| --- | --- | --- | --- | --- | --- |
| Brazil | 7.21 | 19.6 | 5.3 | 1.41 | **24.9** |
| Costa Rica | 3.91 | 6.05 | 1.69 | 0.85 | **10.9** |
| Dominican Republic | 4.85 | 8.92 | 1.77 | 2.97 | **13** |
| Ecuador | 2.48 | 1.35 | 0.87 | 0.75 | **5.26** |
| Mexico | 3.15 | 1.47 | 0.66 | 0.24 | **5.39** |
| Panama | 3.16 | 4.94 | 1.3 | 1.52 | **8.22** |
| Trinidad & Tobago | 2.11 | 1.73 | 0.52 | 0.66 | **4.46** |
| Uruguay | 6.73 | 10.2 | 3.89 | 2.86 | **17** |

*Note: Authors’ estimations. The table reproduces data presented in Table 2 and Table S4.*

***Graph: Disability Prevalence Rates by Type of Disability (Ages 3 and older)***

|  | Motor | Vision | Hearing | Cognitive | Overall |
| --- | --- | --- | --- | --- | --- |
| Brazil | 7.21 | 19.6 | 5.3 | 1.41 | 24.9 |
| Costa Rica | 3.91 | 6.05 | 1.69 | 0.85 | 10.9 |
| Dominican Republic | 4.85 | 8.92 | 1.77 | 2.97 | 13 |
| Ecuador | 2.48 | 1.35 | 0.87 | 0.75 | 5.26 |
| Mexico | 3.15 | 1.47 | 0.66 | 0.24 | 5.39 |
| Panama | 3.16 | 4.94 | 1.3 | 1.52 | 8.22 |
| Trinidad & Tobago | 2.11 | 1.73 | 0.52 | 0.66 | 4.46 |
| Uruguay | 6.73 | 10.2 | 3.89 | 2.86 | 17 |

*Note: Authors’ estimations. The figures reproduce data presented in Table 2 and Table S4.*

**In Table 4**

- In this case, the type of disability seems particularly relevant. What would happen if cognitive disabilities are excluded from the setting of the dependent variable? This can also apply to Table 3.

*Thank you very much for this question. We have included an additional table (Table S11 in the supporting information) estimating the disability prevalence rates and education gaps for the four different types of disabilities. As you correctly anticipated, we observe that disability gaps in school attendance vary between different types of disabilities. The average gap is larger for children with cognitive and motor disabilities, followed by those with hearing disabilities. In children with vision disabilities, we observe a positive but smaller gap among primary school-age children and no systematic gap among secondary school-age children. The patterns are similar when comparing school completion gaps. We have included this discussion in the paper.*

- How do employment gaps correlate with educational gaps? In other words, can’t employment gaps stem from educational gaps? Again, the absence of a conceptual framework it does not allow interpret these results.

*Thank you very much for this question. Education and employment are two closely related outcomes (simultaneously affected by various factors), and we do not have suitable instrumental variables to establish a causal relationship between them.*

*Nevertheless, in response to this comment and that of another reviewer, we have included an additional table (Table S13 in the supporting information) estimating the disability prevalence rates and employment gaps for the four different types of disabilities: vision, motor, hearing, and cognitive. We observe that disability gaps in employment across types of disability are correlated with gaps in education. The average gap is largest for individuals with cognitive disabilities, followed by those with motor and hearing disabilities. In the case of persons with vision disabilities, employment gaps are still positive and large, but smaller than for the other types of disability. We have included this discussion in the paper, which we believe strengthens the link between employment and educational gaps*
